# Longitudinal associations between physical activity and cognition post-stroke: the Nor-COAST study

**DOI:** 10.1093/braincomms/fcag367

**Published:** 2026-10-12

**Authors:** Geske Luzum, Stina Aam, Heather G Allore, Ingvild Saltvedt, Asta K Håberg, Torunn Askim

**Affiliations:** Department of Neuromedicine and Movement Science, Faculty of Medicine and Health Science, NTNU-Norwegian University of Science and Technology, Trondheim 7491, Norway; Department of Neuromedicine and Movement Science, Faculty of Medicine and Health Science, NTNU-Norwegian University of Science and Technology, Trondheim 7491, Norway; Department of Geriatric Medicine, Clinic of Medicine, St. Olavs Hospital, Trondheim University Hospital, Trondheim 7030, Norway; School of Medicine, Yale University, New Haven, CT 06510, United States; Department of Neuromedicine and Movement Science, Faculty of Medicine and Health Science, NTNU-Norwegian University of Science and Technology, Trondheim 7491, Norway; Department of Geriatric Medicine, Clinic of Medicine, St. Olavs Hospital, Trondheim University Hospital, Trondheim 7030, Norway; Department of Neuromedicine and Movement Science, Faculty of Medicine and Health Science, NTNU-Norwegian University of Science and Technology, Trondheim 7491, Norway; Department of Neuromedicine and Movement Science, Faculty of Medicine and Health Science, NTNU-Norwegian University of Science and Technology, Trondheim 7491, Norway

**Keywords:** stroke, cognition, step count, physical activity, secondary prevention

## Abstract

Previous research has been inconsistent regarding the longitudinal relationship between physical activity and cognition after a stroke. In this study, we hypothesized that (i) higher levels of objectively measured daily step counts would be associated with better cognitive function at a group level (between-person level), and (ii) at an individual level, changes from expected step counts would be bi-directionally associated with changes from expected cognitive performance (within-person level) at the same and subsequent time points. We used data from 468 participants of the Norwegian Cognitive Impairment after Stroke study (Nor-COAST), a large multicentre cohort study with follow-up at 3, 18 and 36 months post-stroke. At each follow-up, average step count over 7 days was recorded using accelerometers, and cognitive performance was assessed with the Montreal Cognitive Assessment (MoCA), the Trail Making Test A and B (TMT-A, TMT-B), and the Word List Memory and Recall Test (delayed recall). Adjusted random intercept cross-lagged panel models (RI-CLPMs) were implemented. One RI-CLPM was built for each cognitive outcome, separating between- from within-person associations. To balance baseline characteristics of included and excluded participants in Nor-COAST and to account for selection bias, inverse probability weighting was performed using age, sex, and baseline functional independence. Between-persons, we found a time-invariant association between step count and TMT-A (*β* = 0.24, 95% CI: 0.09 to 0.40) and TMT-B (*β* = 0.22, 95% CI: 0.09 to 0.34); after correcting for multiple comparison the association with MoCA was not significant (*β* = 0.14, 95% CI: 0.01 to 0.28), and there was no association with the Word List Memory and Recall Test (*β* = 0.09, 95% CI: −0.04 to 0.22). At the within-person level, we observed neither cross-sectional nor cross-lagged associations between step count and any of the cognitive tests. The RI-CLPM fitted the data appropriately and had robust testing models with and without inverse probability weighting. After a stroke, people who, on average, took more steps tended to also perform better on TMT-A and TMT-B scores. However, at an individual level, a person who walked more (or had better cognitive scores) than expected did not have a higher likelihood of also performing better on cognitive tests (or walking more) than their expected average, respectively. Therefore, the observed association between step count and cognitive function after stroke might be explained by consistent individual differences over time, rather than by temporary changes within an individual's typical level of physical activity or cognition.

## Teaser

Luzum *et al*. report that following stroke, people who generally walked more each day performed better on tests of complex attention and executive function, but not global cognition and memory. Over time, changes in walking didn’t lead to changes in cognitive abilities, and changes in cognitive abilities didn’t affect walking.

## Graphical Abstract

**Graphical Abstract:**
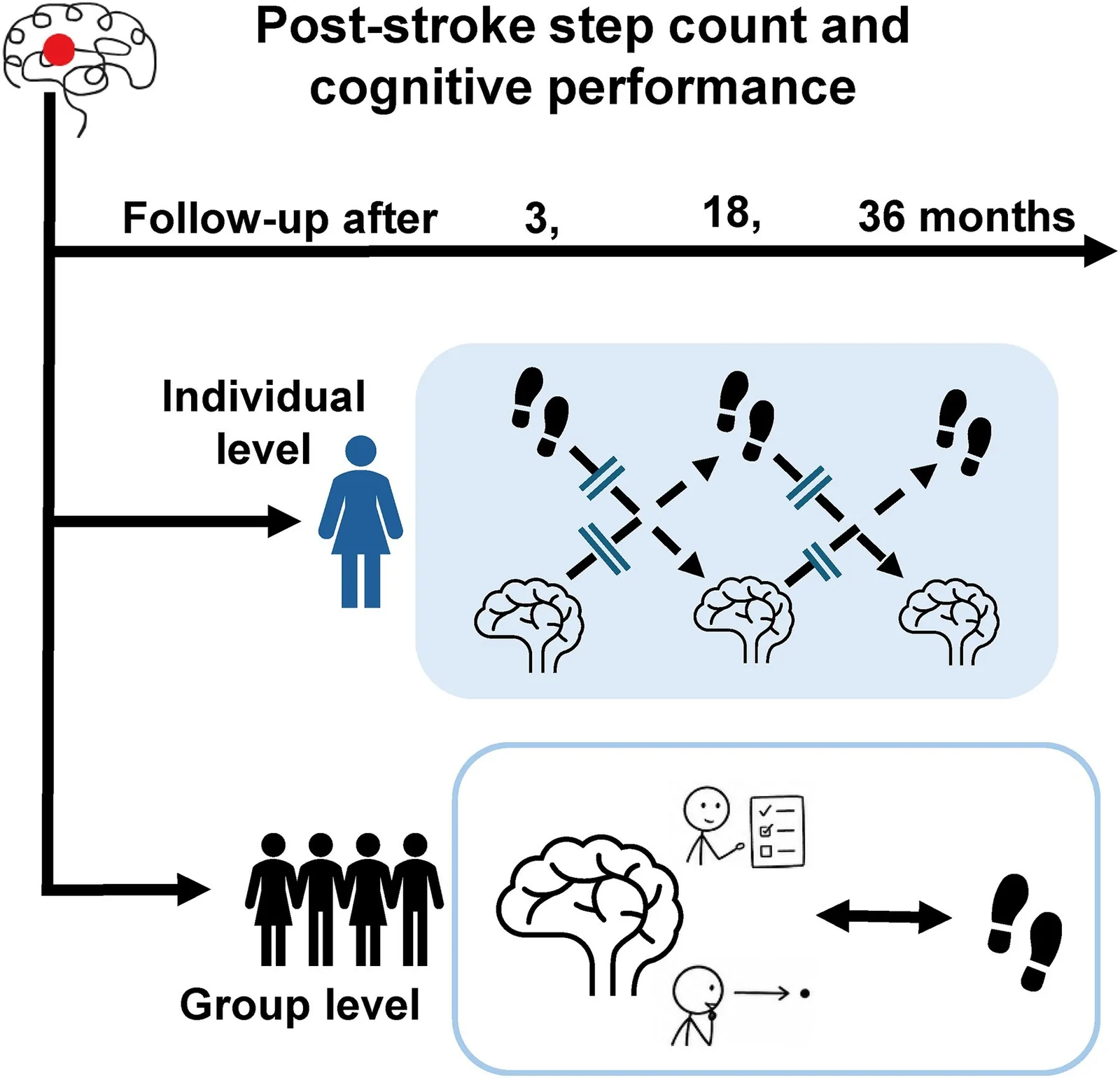

## Introduction

Cognitive impairment is common after stroke, affecting around 4 out of 10 people, even though most strokes are classified as mild.^1-3^ The stroke event itself has been linked to an immediate loss of cognition, followed by an accelerated decline over time, compared with the pre-stroke cognitive trajectory.^4,5^ Individual cognitive trajectories during the first year after stroke are highly heterogeneous. Some people experience accelerated decline, others remain stable, or recover to their pre-stroke levels.^6,7^ Longitudinal data show that even when there is significant improvement within the first 3 months of recovery, half of stroke survivors are at risk of cognitive decline 1 year after the stroke.^8^

Stroke and cognitive impairment share several risk factors, such as lower education, hypertension, heart disease, social isolation, smoking, alcohol consumption, loneliness, and physical inactivity.^6,9-11^ While we know that midlife is an important window of risk exposure for later-life cognitive function for most of these factors, including physical activity,^10,12^ it has been shown that being physically active at any time in adulthood, and to any extent, could be linked with a higher later-life cognitive state.^13^

### Physical activity as a determinant of cognitive performance

Emerging evidence supports the role of physical activity in mitigating cognitive decline and reducing the risk of dementia (Livingston, Huntley *et al*. 2024). In older people, it was shown that individuals who decreased their peak oxygen uptake over 5 years had higher odds of mild cognitive impairment compared with those who maintained their uptake, suggesting that physical activity, particularly that which affects cardiorespiratory fitness, may influence the association between physical activity and cognition.^14^ For individuals in all phases after stroke, there is evidence of an overall positive effect of physical activity interventions on attention and processing speed.^15,16^ A prospective longitudinal study following people with mild strokes suggests that global cognition scores during the first year post-stroke were consistently associated with non-modifiable factors, such as age and lesion side, and could only uncover one modifiable factor: physical activity.^8^

### Cognitive performance as a determinant of physical activity

Yet, reduced post-stroke cognitive function may be a barrier to engaging in physical activity,^17^ for instance, due to difficulties in planning and understanding the importance of physical activity for secondary prevention.^18^ A cross-sectional study further demonstrated that motor or cognitive impairments after stroke were common (30–40%), with the majority of them having concurrent impairments across cognitive domains and physical capacity outcomes,^19^ likely affecting their gait. Findings from a longitudinal analysis spanning 28 years, which observed participants from midlife to old age, found that until 10 years before dementia diagnosis, self-reported physical activity trajectories of those who would eventually be diagnosed with dementia did not differ significantly from the trajectories of individuals who were not. However, starting 9 years before diagnosis, the physical activity levels of individuals who would experience dementia started to be lower than those of individuals who did not experience dementia.^20^ In line with this, it has been shown that better global cognitive function in the acute phase after a stroke was associated with higher moderate to vigorous PA levels and step count 2 years later.^18^ Using a cross-sectional design, lower global cognition after stroke has been linked to increased odds of not meeting physical activity guidelines, with physical capacity mediating that association.^21^

### Bi-directionality

Taken together, the link between physical activity and cognitive function is not fully understood, but it is likely bidirectional and varies across different cognitive domains.^10,22^ While consistent findings in the literature relate to the positive effects of physical activity on executive function^23-25^ one should be cautious about interpreting this as the most important, since many studies tend to prioritize the assessment of executive functions over other cognitive domains. For instance, the cognitive domains of language, visuospatial performance, and memory have been underrepresented in the literature.^15^ A particularly robust association between post-stroke physical activity and executive function has not been previously demonstrated in the stroke cohort.^15,26,27^ Additionally, there is considerable variability in the types and quality of instruments used to test both cognitive domains and physical activity.^24,27,28^

Given that step count is a reliable measure related to functional outcomes in older adults that can easily be captured in clinical and non-clinical settings, this study focused on step count as a physical activity variable.^29,30^ To address current knowledge gaps^18,31^ on bi-directionality and the association between physical activity and cognitive function after stroke, this observational 3-year cohort study aimed to investigate the relationship between average daily step count and cognitive performance across multiple domains following stroke. Our research question was whether there was an association between average daily step count after stroke and global cognitive function, memory, executive function, and attention, detectable at both the between- and within-person level at 3, 18 and 36 months post-stroke. We hypothesized that higher levels of objectively measured average daily step counts would be differentially associated with improved global cognition, complex attention, executive function and memory, at the between-person level. Additionally, we hypothesized that changes in individual step counts would be bidirectionally associated with changes in cognitive performance at the same and later time points at the within-person level.

## Materials and methods

### Design and participants

This study employs a longitudinal multicentre cohort observational design. Data were from the Norwegian Cognitive Impairment After Stroke study (Nor-COAST), which includes 815 participants with acute first-ever and recurrent strokes, diagnosed according to the World Health Organization (WHO) criteria or with findings of acute brain infarction or intracerebral haemorrhage on neuroimaging.^32^

From May 2015 through March 2017, participants were eligible for inclusion in Nor-COAST if they were admitted to one of the five participating hospitals in Norway within 7 days after symptom onset, were residing in the catchment areas of the recruiting hospitals, were Scandinavian-speaking, and 18 years or older. The exclusion criterion was an expected survival of <3 months.^32^ To be included in this sub-study, participants additionally had to have (i) at least one valid accelerometry measure of physical activity over at least three consecutive days at the follow-ups at 3, 18 or 36 months post-stroke, (ii) at least one valid measure of Montreal Cognitive Assessment (MoCA), the Word List Memory and Recall Test (WLMT delayed recall test), Trail Making Test A (TMT-A) and Trail Making Test B (TMT-B). Further, participants with a diagnosis of dementia at baseline (*N* = 8) or no information on stroke severity (*N* = 8) were excluded from this study.

Participants’ consent was obtained in accordance with the Declaration of Helsinki. The regional committees for medical and health research (REK) granted ethical approval (REK number: 2015/171; 2017/1415). Participants provided written informed consent prior to their inclusion in the study. In cases where they were unable to give consent, they were included if proxies did not decline participation (passive proxy-based informed consent). This is in line with the Norwegian regulations for patients who are unable to give oral or written consent themselves.

### Data collection

The analyses in the present study utilized data from 468 (57.4%) of the 815 Nor-COAST participants. The inclusion and attrition of participants are presented in the flowchart in Fig. 1.

**Figure 1:**
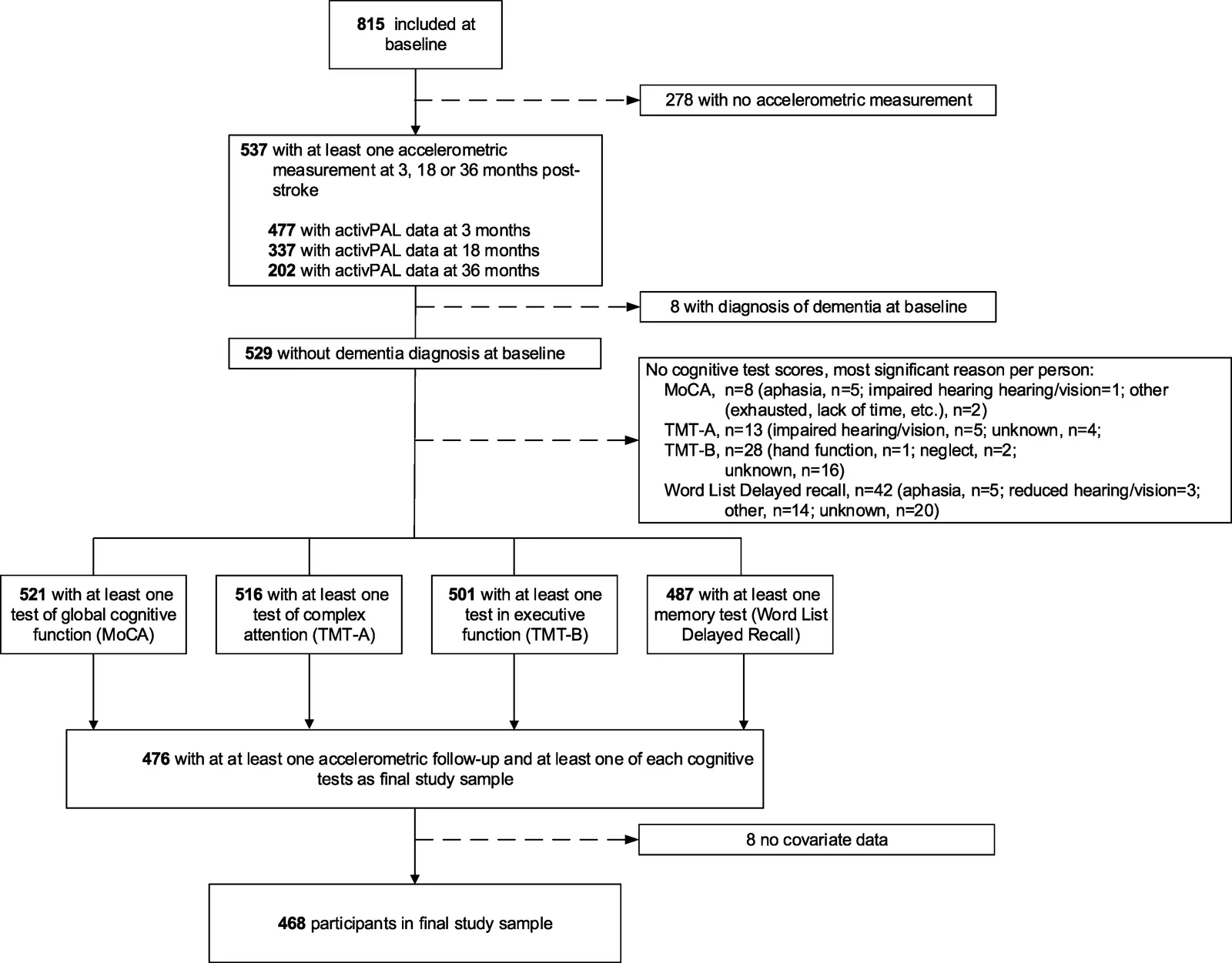
**Flow of participants**. MoCA, Montreal Cognitive Assessment; TMT-A, Trail Making Test A; TMT-B, Trail Making Test B.

Baseline assessments were performed during the hospital stay at discharge or on the seventh day for participants with longer stays. Follow-up assessments were conducted at an outpatient clinic at 3, 18 and 36 months post-stroke. Trained research assistants carried out all assessments (and test batteries).

Sociodemographic and health data (age, sex, years of education, living situation, body mass index (BMI) at the time of stroke, and diabetes mellitus or dementia before the stroke) were obtained from medical records and standardized questionnaires for patients and/or caregivers. Stroke severity was evaluated using the National Institutes of Health Stroke Scale (NIHSS, score: 0–42), measured on the day of admission to the hospital (NIHSS-0) and one day after admission (NIHSS-1). An NIHSS score below six was considered a mild stroke.^2,33^ The aetiology of the stroke was determined using the TOAST criteria.(Adams, Davis *et al*. 1999) Functional independence in activities of daily living (ADL) was assessed at baseline and at 3-month follow-up using the modified Rankin Scale (mRS). The mRS is a score from zero to six, with higher scores indicating greater dependence in ADLs, while the sixth category indicates death.(Wolfe, Taub *et al*. 1991) Symptoms of anxiety and depression were evaluated using the Hospital Anxiety and Depression Scale (HADS). HADS comprises 14 items, each scored from zero (no symptoms) to three.^34^ Data on objective physical activity behaviour and cognitive functions were collected at each of the three follow-up time points of the study.

### Accelerometry at 3, 18 and 36 months post-stroke

The average daily step count was recorded using an activPAL accelerometer at 3, 18 and 36 months post-stroke.^35^ At each follow-up, the accelerometer was attached to the front of the participants’ unaffected thighs. Each accelerometer follow-up lasted for 7 consecutive days. Participants were instructed not to remove the sensor, even while showering or sleeping. A semi-automated algorithm was employed to distinguish wake time from sleep time.^36^ Accelerometer measurements were deemed valid if data were available from at least 3 consecutive measurement days per follow-up period. Details regarding the collection and processing of accelerometric data are described elsewhere.^37^

### Cognitive test battery at 3, 18 and 36 months post-stroke

The neurocognitive domains were defined according to the DSM-5 criteria.^38^ The cognitive test battery consisted of MoCA as a measure of global cognitive function, the TMT-A as a measure of complex attention, the TMT-B as a measure of executive function, and the WLMT delayed recall test, from the Consortium to Establish a Registry of Alzheimer’s Disease (CERAD) as a measure of memory.^39-42^ MoCA version 7.3 was used at the 3-month follow-up, and version 7.1 was used at the 18- and 36-month follow-ups. In cases where participants started but did not complete the TMT-A and TMT-B due to cognitive impairment, the test results were recorded as equal to the time of interruption of the tests, the maximum values at 180 and 300 s, respectively.^43^ For TMT-A, 24 scores were set to 180 s, and for TMT-B, 160 scores were set to 300 s across all follow-ups combined. The raw scores of all cognitive tests were demographically adjusted (*z*-scores) based on normative data as described in detail in the Supplementary material (Supplementary material: ‘Normalization of cognitive test scores’ and Supplementary Table 1). Negative values *z*-scores for all cognitive domains indicate performance below the normative average.

### Statistical analyses

We employed adjusted standardized random intercept cross-lagged panel models (RI-CLPMs) with three time points to investigate the bidirectional associations between average daily step count and the four normalized cognitive outcomes: global cognitive function, complex attention, executive function, and memory.^44^ The RI-CLPM is a structural equation modelling approach to longitudinal data. As a central difference to the cross-lagged panel model (CLPM), it separates within-person dynamics from stable between-person differences, thereby helping to explore bidirectionality and temporal ordering.^45^ The distinction between within- and between-person associations assumes that each individual has a specific, relatively stable average level of physical activity and cognitive performance across follow-ups (captured by the random intercepts), around which their measurements dynamically change over time.^45^

One RI-CLPM was built for each cognitive outcome (Fig. 2). Person-centred latent variables were created for each observed measure of average daily step count and for each cognitive outcome at each time point, with each loading constrained to 1. This resulted in six within-person latent factors. Two additional latent variables captured stable between-person differences (random intercept) in average daily step count (kappa) and cognitive function outcomes (omega). Given the slightly unequal time intervals between follow-ups (15 months between the first and second follow-ups versus 18 months between the second and third follow-ups), we specified that correlations and cross-lagged effects were allowed to vary across time in all models. Sex, age, education, living situation (alone/with someone), and stroke severity were added as covariates and controlled for at the random-intercept level. The choice of covariates was based on prior literature identifying potential confounders, as our aim was to establish associations rather than predict outcomes. The number of confounders was limited by considerations of model complexity. Supplementary Table 2 compares included versus excluded participants. Inverse probability weighting (IPW) was employed to address concerns of selection bias and adjust for the probability of inclusion in this study. IPW was based on the variables age, sex, and functional independence proxied by mRS (see details in Supplementary Table 5, Supplementary Figs 2 and 3). Weights were trimmed at the 97th percentile.

**Figure 2:**
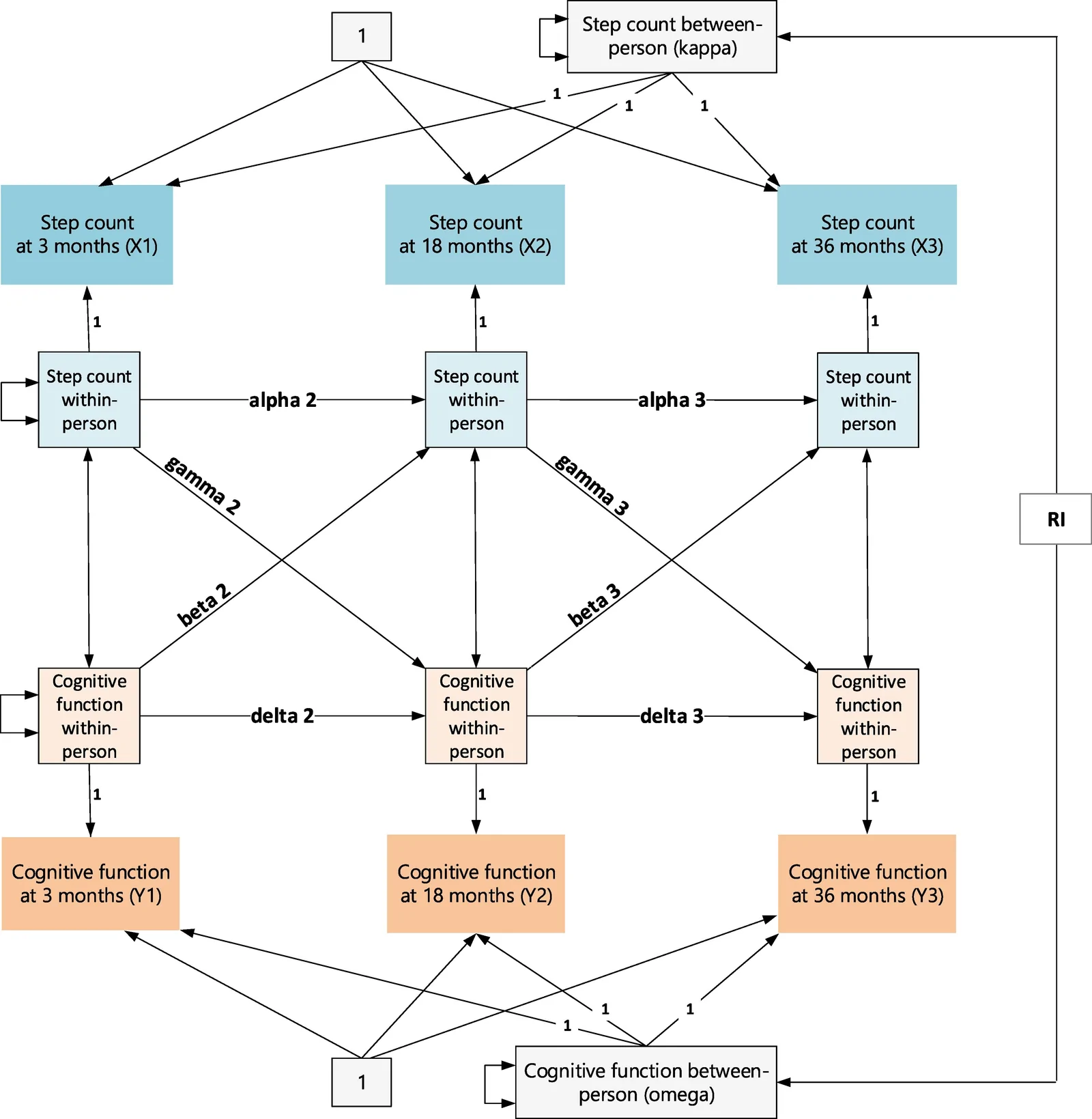
**Random intercept cross-lagged panel model (RI-CLMP) specifications**. The Figure shows the model specifications for the RI-CLPM used to examine the longitudinal bidirectional associations between average daily step count and the four cognitive outcomes: global cognitive function, complex attention, executive function and memory. The model was adjusted for sex, age, education, living situation, depressive symptoms and stroke severity at the random intercept level (kappa and omega). X_1–3_ and Y_1–3_ denote the observed variables measured with accelerometry over 1 week and the cognitive test battery at each test time point (3 months, 18 months and 36 months post-stroke), respectively. The auto-regressive pathways alpha and delta capture within-person carry-over effects. They can be interpreted as indicating the extent to which prior differences from their expected scores predict within-person deviations at subsequent measurement time points. The cross-lagged paths gamma and beta can be interpreted as the within-person effect of one variable on the subsequent measure of the other variable. The cross-sectional paths represent correlations between the latent variables of X_1_ and Y_1_ at 3 months post-stroke, and correlated changes between the latent variables of X2 & Y2, and X3 & Y3, at 18 and 36 months, respectively.

Standardized beta (β) coefficients were presented to compare the relative strength of the association between pathways within the same models.^46^ Additionally, unstandardized coefficients (B) were estimated, and both were considered for the interpretation of results. To address incomplete data, we utilized the case-wise maximum likelihood estimation method with missing values. We assumed data to be missing at random, conditional on the observed covariates included in our models, which are known predictors of both attrition and the variables of interest. Model fit indices of the final adjusted and weighted models were employed to assess goodness of fit (see Supplementary Table 3). Several sensitivity analyses were conducted. All models were re-run with covariates included at the level of the observed variables to test the robustness of the results. Additional sensitivity analyses with 41% drop out, setting power to 0.8 and alpha at 0.5 or alpha at 0.0125 had detectable effect sizes of 0.042 and 0.029, respectively. In addition, the Holm-Bonferroni method was used to test the robustness of results when correcting for multiple comparisons.^47^ The threshold for statistical significance was a *P*-value of < 0.05. Statistical analyses were conducted using STATA Version 18.0 software (StataCorp, College Station, TX, USA) and RStudio (RStudio 2024.04.1), utilizing the lavaan package.^48^

## Results

Figure 1 shows the flow of all 468 participants included in the analysis. Table 1 shows the characteristics of the study participants. At baseline, participants had a mean age of 70.9 years (SD: 11.4), and 41.2% were women. Overall, participants had high education levels (>12 years), and most experienced mild strokes [NIHSS-1, mean (SD): 2.5 (3.6)]. Supplementary Table 6 presents a comparison of the characteristics of our study participants and the overall Nor-COAST sample before and after weighing. Supplementary Fig. 3 shows that covariate balance improved after weighting, with all but one variable falling below the commonly used threshold of good balance (≤0.10).^49^

**Table 1:** Participant characteristics

CHARACTERISTICS	*N* = 468
Sex (self-report)	
Female, *n* (%)	193 (41.24)
Male, *n* (%)	275 (58.76)
Age, years, mean (SD)	70.86 (11.44)
Age range	[33 to 96]
Education, years, mean (SD)	12.85 (3.69)
TOAST, *n* (%)	
Large vessel disease	44 (10.68)
Cardioembolism	86 (20.87)
Small vessel disease	108 (26.21)
Other aetiology	11 (2.67)
Undetermined/multiple possible causes	163 (39.56)
Living alone, *n* (%)	145 (30.98)
Previous stroke or TIA	100 (21.4)
mRS at baseline, mean (SD)	1.92 (1.21)
mRS at 3 months, mean (SD)	1.42 (0.95)
mRS at 3 months, *n* (%)	
No symptoms (mRS = 0)	79 (17.06)
No significant disability (mRS = 1)	171 (36.93)
Slight disability (mRS = 2)	162 (34.99)
Moderate disability (mRS = 3)	40 (8.64)
Moderate to severe disability (mRS = 4)	11 (2.38)
Diabetes mellitus *n* (%)	93 (19.87)
BMI, mean (SD)	26.23 (4.15)
NIHSS-1, mean (SD)	2.47 (3.60)
Median (25th to 75th percentile)	1 (0 to 3)
Lesion type, *n* (%)	
Ischemic stroke	427 (91.24)
Haemorrhagic stroke	41 (8.76)
HADS at 3 months, mean (SD)	7.00 (6.00)

Deviating sample size due to missingness: BMI (*N* = 449), mRS at baseline (*N* = 466), mRS at 3 months (*N* = 463), TOAST (*N* = 412), HADS (*N* = 410).

BMI, body mass index; HADS, hospital anxiety and depression scale; mRS, modified rankin scale; NIHSS-1, National institutes of health stroke scale measured 1 day after hospital admission; TIA, transient ischemic attack; TOAST, categorization of subtypes of ischemic stroke developed for the trial of org 10172 in acute stroke treatment.

Table 2 presents the accelerometric measurements of average daily step counts, along with the raw and age-, sex-, and education-specific normative scores for the four cognitive tests across the three follow-ups. The average duration of the accelerometric measurement across follow-ups was 5.7 consecutive days (SD: 0.7). The number of daily steps remained relatively stable across follow-ups, although considerable variation was observed.

**Table 2:** Average daily step count and cognitive test scores over time

Variables	Follow-up after stroke					
	3 months	*N*	18 months	*N*	36 months	*N*
Average daily step count	2899 (1776)	416	2438 (1498)	314	2882 (1769)	195
MoCA	24.90 (3.98)	457	25.10 (4.09)	400	25.87 (3.89)	270
*z*-score MoCA	−0.44 (1.76)	457	−0.32 (1.77)	400	0.06 (1.74)	270
TMT-A	57.35 (38.15)	456	52.36 (32.26)	390	53.75 (35.79)	269
*z*-score TMT-A	−0.64 (1.28)	456	−0.43 (1.21)	390	−0.45 (1.26)	269
TMT-B	153.71 (83.41)	450	142.35 (79.10)	382	132.67 (76.56)	257
*z*-score TMT-B	−1.04 (1.31)	450	−0.86 (1.30)	382	−0.73 (1.30)	257
Delayed recall	5.36 (2.60)	430	5.35 (2.61)	333	5.73 (2.57)	232
*z*-score delayed recall	−0.06 (1.60)	430	0.07 (1.63)	333	0.34 (1.62)	232

The Table displays the average daily step count, cognitive scores, and standardized cognitive test scores [mean (SD)] of participants across time. The standardized scores indicate how much the study sample’s (*N* = 468) average cognitive scores differ from the expected (normative) average scores for individuals of the same sex, age, and educational level. The units are measured in standard deviations from the normative mean (zero). The Montreal Cognitive Assessment (MoCA) score ranges from 0 to 30; Trail Making Test A (TMT-A) and Trail Making Test B (TMT-B) are timed in seconds; the Delayed Recall 10-word test shows the number of words remembered.

All group cognitive test scores showed an average performance below what would be expected for individuals of the same age, sex and education level (Table 2) at the 3-month follow-up. Average global cognition (MOCA) improved over time, initially below the normative mean performance at 3 months, but reaching the average typical level by 36 months. For complex attention (TMT-A *z*-score), participants consistently scored below the normative mean across all three follow-ups, with scores increasing over time. Among all the cognitive tests, the lowest performance was observed in executive function (TMT-B *z*-score). TMT-B *z*-scores were up to 1 SD below the normative mean at 3 months, but, as with the other tests, scores increased at later follow-ups to −0.86 (SD: 1.30) and −0.73 (SD: 1.30) SDs below the normative mean at 18 and 36 months, respectively. The average score on the WLMT delayed recall test was similar across all follow-ups, with *z*-scores ranging from −0.06 to 0.34, indicating performance that matched the expectation in the normative sample.

### Within and between-person associations of step-count and cognitive outcomes

Supplementary Table 7 provides all details of each of the RI-CLPMs, including unstandardized and standardized estimates.

### Steps and MoCA

The associations between step count and global cognition (MoCA *z*-score) after stroke, divided into within- and between-person effects, are illustrated in Fig. 3. The overall model fit of the adjusted and weighted RI-CLPM for average daily step count and MoCA *z*-scores was good (Supplementary Table 3). At the between-person level, we observed a positive association between step count and MoCA *z*-scores (standardized *β* of kappa and omega: *β* = 0.14, 95% CI: 0.01 to 0.28, *P* = 0.04), with participants having higher step counts across the three measurement time points generally also performing better on the MoCA *z*-scores. This Random Intercept association was, however, no longer significant in the unstandardized model (*P* = 0.06) and also insignificant after Holm-Bonferroni correction. At the within-person level, the cross-sectional pathways were not associated. The autoregressive paths for both step count and MoCA *z*-scores were not significant in the standardized output, meaning that within-person deviations from expected scores (i.e. predicted means in step count and MoCA *z*-scores) did not predict changes at subsequent follow-up. While Supplementary Table 7 indicates an association of the unstandardized autoregressive pathways alpha2, delta3, both the standardized estimates of these pathways, as well as the Holm-Bonferroni corrections, showed these associations not to be robust. Cross-lagged within-person paths from step count to MoCA *z*-scores and from MoCA *z*-scores to step count were not significantly associated at any follow-up.

**Figure 3:**
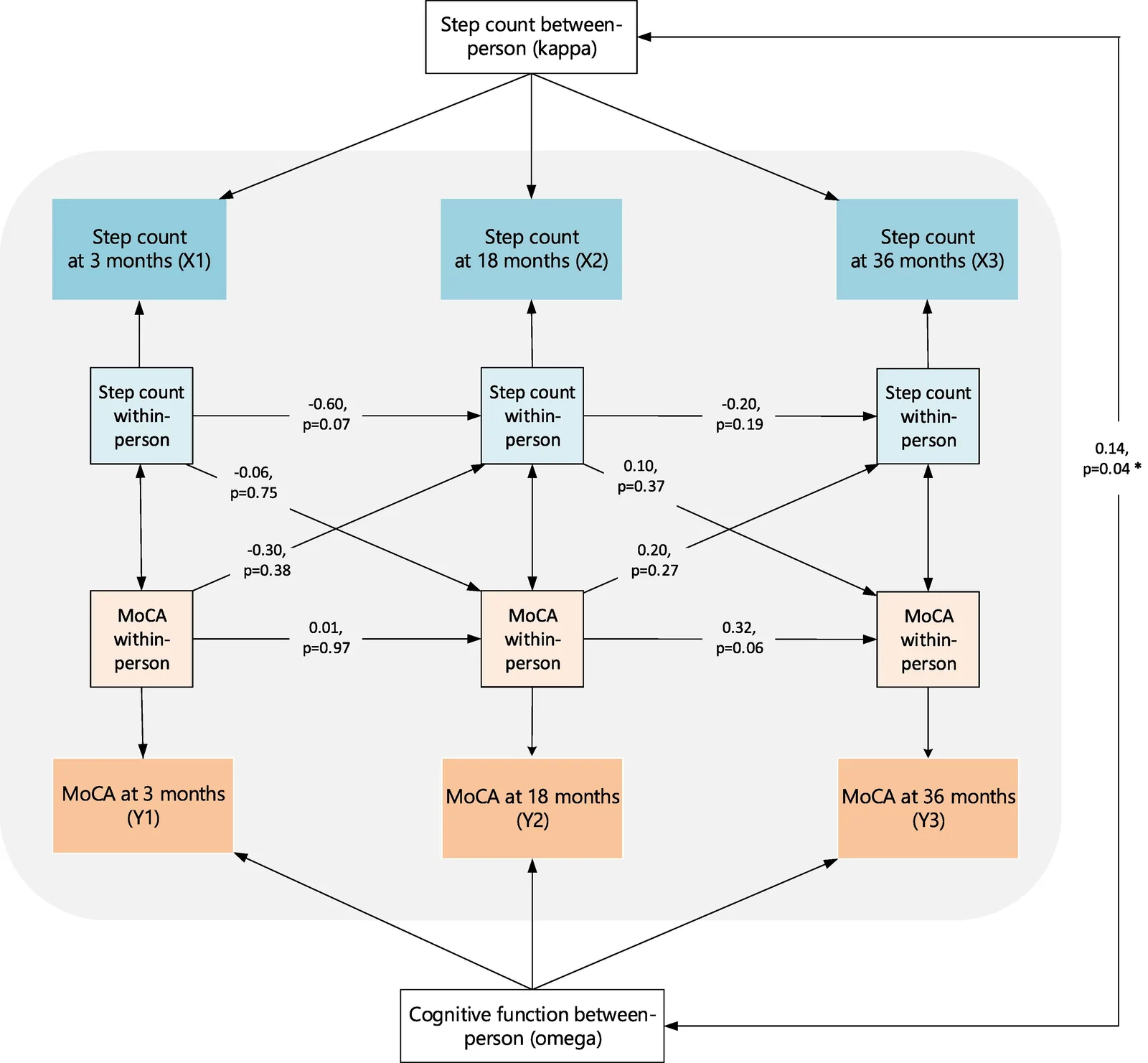
**RI-CLPM of step count and global cognitive function (MoCA) post-stroke**. Standardized, adjusted random intercept cross-lagged panel model (RI-CLPM): * *P* < 0.05; ** *P* < 0.01; *** *P* < 0.001. In total, 468 participants were included, who contributed accelerometer data with *N* = 416, *N* = 314, and *N* = 195; and MoCA data with *N* = 457, *N* = 400, and *N* = 270, at 3, 18 and 36 months post-stroke respectively. Between-person differences are captured by the random intercept (kappa-omega, outside the grey box). All within-person pathways are displayed in the grey box. Note that after Holm-Bonferroni correction done as a sensitivity analysis, none of the between- or within-person pathways remained below the significance level. MoCA, Montreal Cognitive Assessment.

### Steps and TMT-A

The associations between step count and complex attention (TMT-A z-score) after stroke, divided into within- and between-person effects, are illustrated in Fig. 4. The overall model fit (of the RI-CLPM) on average daily step count and TMT-A *z*-scores was good (Supplementary Table 3). At the between-person level, a positive association was found between step count and TMT-A *z*-scores (*β* = 0.24, 95% CI: 0.09 to 0.40, *P* = 0.002), indicating that participants with higher step counts across all follow-ups generally also scored better on the TMT-A. This finding was robust, even after Holm-Bonferroni correction. At the within-person level, there were no significant cross-sectional or cross-lagged associations between average daily step count and TMT-A *z*-scores, so that changes from the expected step count were not associated with deviations from the individually expected TMT-A scores at any follow-up, or vice versa. As observed for the MoCA *z*-scores, the autoregressive pathways were not significant, except for alpha2 (step count from 3 to 18 months), which was borderline significant in the unstandardized estimates, but not after correction for multiple comparisons.

**Figure 4:**
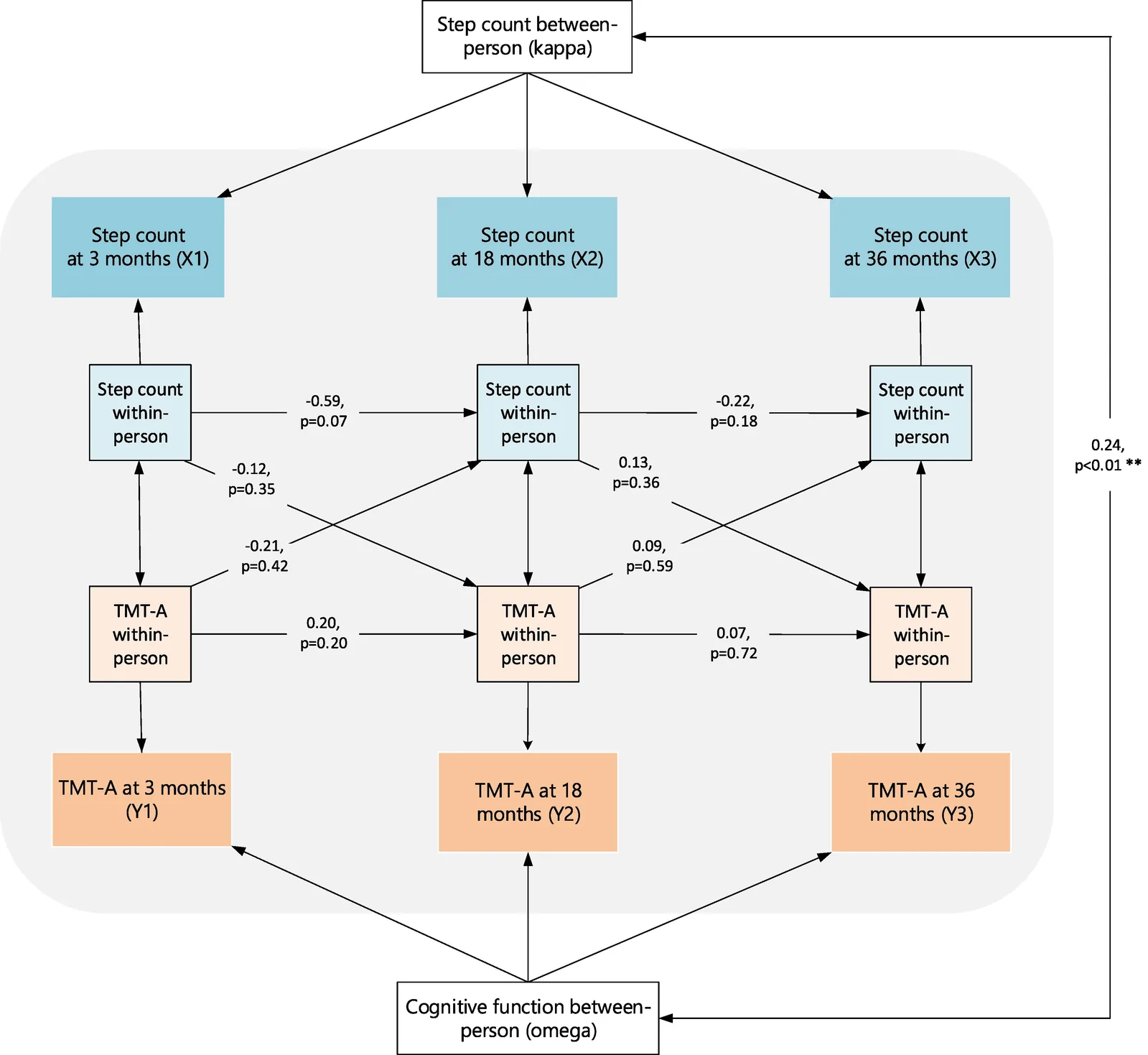
**RI-CLPM of step count and complex attention (TMT-A) post-stroke**. Standardized, adjusted random intercept cross-lagged panel model (RI-CLPM): * *P* < 0.05; ** *P* < 0.01; *** *P* < 0.001. Between-person differences are captured by the random intercept (kappa-omega, outside the grey box). All within-person pathways are displayed in the grey box. In total, 468 participants were included, who contributed accelerometer data with *N* = 416, *N* = 314, and *N* = 195; and TMT-A data with *N* = 456, *N* = 390, and *N* = 269, at 3, 18 and 36 months post-stroke respectively. Sensitivity analyses using Holm–Bonferroni correction yielded consistent results, supporting the robustness of the findings. TMT-A, Trail Making Test A.

### Steps and TMT-B

The associations between step count and executive function (TMT-B *z*-score) after stroke, divided into within- and between-person effects, are illustrated in Fig. 5. The overall model fit of the RI-CLPM on average daily step count and executive function was good (Supplementary Table 3). At the between-person level, a positive association was found between step count and TMT-B *z*-scores (*β* = 0.22, 95% CI: 0.09 to 0.34, *P* = 0.001), such that higher step counts were generally observed together with better TMT-B *z*-scores. This finding was robust, also after correction for multiple comparisons. At the within-person level, there were no significant cross-sectional associations between average daily step count and TMT-B *z*-scores at any follow-up. Neither the autoregressive paths of average daily step count and TMT-B *z*-scores nor the within-person cross-lagged paths from step count to TMT-B *z*-scores or TMT-B *z*-scores to step count showed significant associations at the follow-ups.

**Figure 5:**
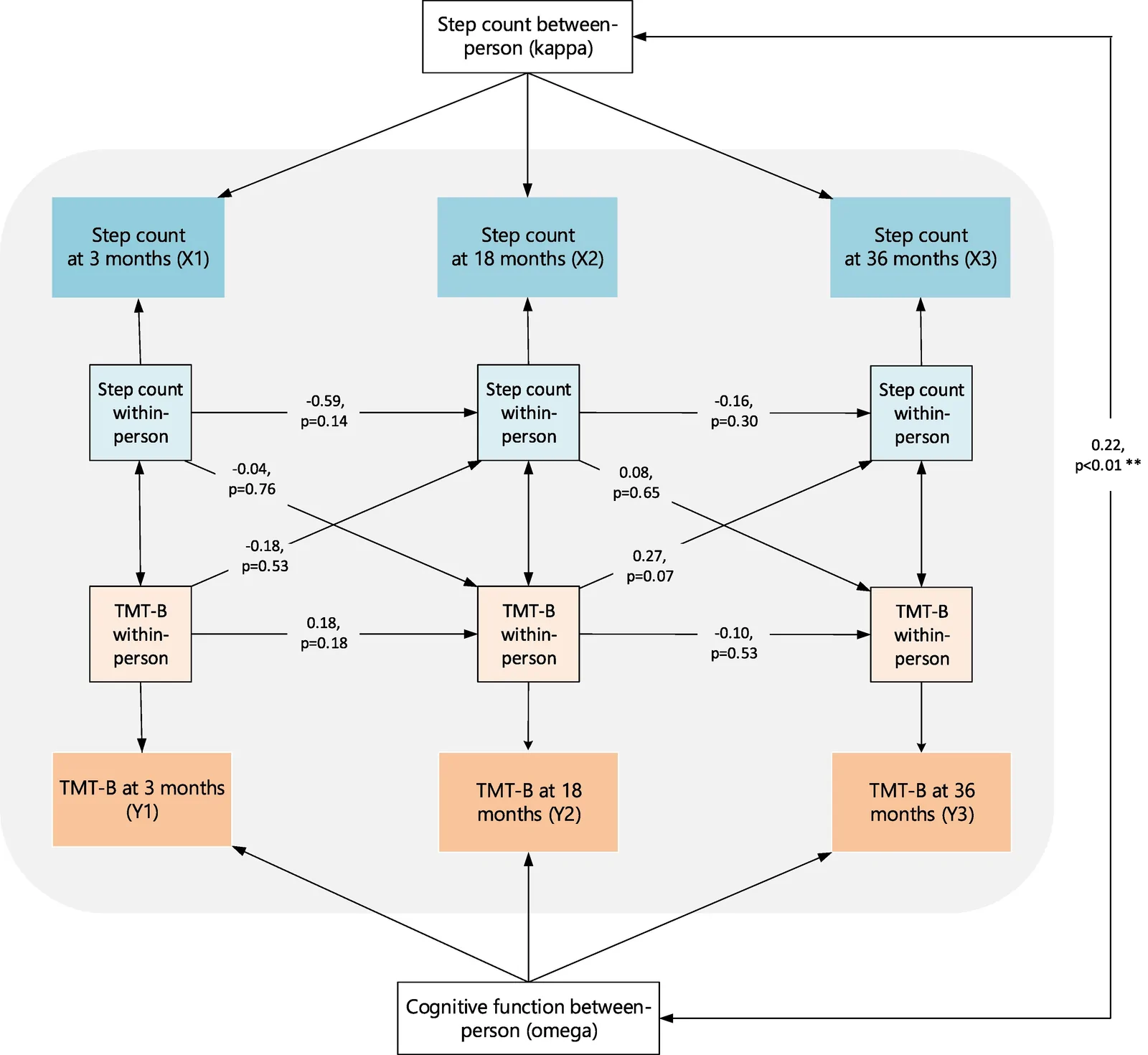
**RI-CLPM of step count and executive function (TMT-B) post-stroke**. Standardized, adjusted random intercept cross-lagged panel model (RI-CLPM): * *P* < 0.05; ** *P* < 0.01; *** *P* < 0.001. Between-person differences are captured by the random intercept (kappa-omega, outside the grey box). All within-person pathways are displayed in the grey box. In total, 468 participants were included, who contributed accelerometer data with *N* = 416, *N* = 314, and *N* = 195; and TMT-B data with *N* = 450, *N* = 382, and *N* = 257, at 3, 18 and 36 months post-stroke respectively. Sensitivity analyses using Holm–Bonferroni correction yielded consistent results, supporting the robustness of the findings. TMT-B, Trail Making Test B.

### Steps and WLMT delayed recall test

The associations between step count and memory (WLMT, delayed recall test *z*-score) after stroke, divided into within- and between-person effects, are illustrated in Fig. 6. The overall model fit of the RI-CLPM regarding the associations between step count and the WLMT delayed recall test *z*-scores was good (Supplementary Table 3). The model revealed no association between step count and the WLMT delayed recall test at the between-person level (kappa & omega). Neither the cross-lagged pathways nor the cross-sectional or autoregressive pathways were significant, except for alpha2, which was borderline significant in the unstandardized estimates (see Supplementary Table 7), but not in the standardized estimates and not after correction for multiple comparisons.

**Figure 6:**
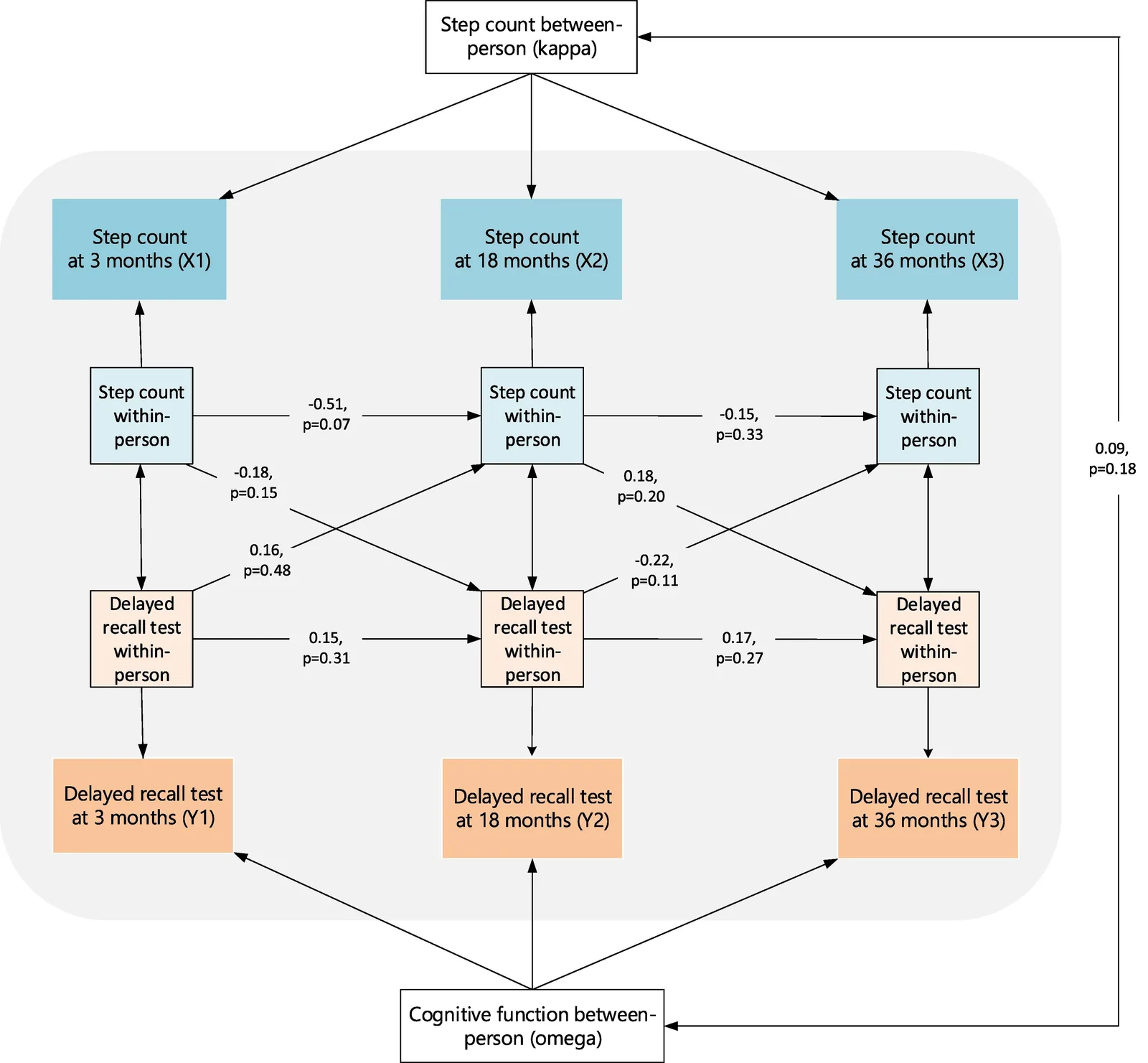
**RI-CLPM of step count and memory (word list memory and recall test, delayed recall) post-stroke**. Standardized, adjusted random intercept cross-lagged panel model (RI-CLPM), *N* = 468: * *P* < 0.05; ** *P* < 0.01; *** *P* < 0.001. Sensitivity analyses using Holm–Bonferroni correction yielded consistent results, supporting the robustness of the findings. Between-person differences are captured by the random intercept (kappa-omega, outside the grey box). All within-person pathways are displayed in the grey box. In total, 468 participants were included, who contributed accelerometer data with *N* = 416, *N* = 314, and *N* = 195; and delayed recall test data with *N* = 430, *N* = 333, and *N* = 232, at 3, 18 and 36 months post-stroke respectively.

### Sensitivity analysis

Only the associations between step count and TMT-A, as well as TMT-A at the random intercept level, were robust to correction for multiple comparisons. The additional inclusion of covariates at the level of the observed variables (within-person level) demonstrated consistent findings with the main models.

## Discussion

In this study, we examined the associations between step count and cognitive domains over time to understand the long-term associations and their direction after a stroke. By separating between-person and within-person associations, we found significant associations between step count and complex attention and executive function at the group level. However, at the individual level, a deviation from a person’s expected step count or cognitive score at one point in time was not associated with a relative change in the other variable at the same or subsequent follow-up times. While our findings remained inconclusive regarding the direction of the associations, they suggest that stroke survivors who generally took more steps also tended to have better performance in complex attention and executive functions. Therefore, the observed association between step count and cognitive function after stroke seems to be explained by consistent individual differences over time, rather than by temporary changes within an individual's typical level of physical activity or cognition.

### Between-person associations of step count and cognition

In this study, higher step counts were differentially associated with various cognitive domains at a between-person level. While we observed a clear association between step count and the cognitive *z*-scores representing complex attention and executive functions at the between-person level, higher step counts were only marginally related to global cognitive function (MoCA-*z*-scores), but did not remain significant after correction for multiple comparisons. Step count after stroke was not associated with memory (delayed recall *z*-score) at the between-person level. Our findings thereby contribute to the inconsistent body of observational studies examining the association between accelerometer-based^18,50-52^ and self-reported physical activity with cognitive function after stroke in either direction.^17,53^

Walking is no longer viewed as a purely automated motor process that requires little higher-level cognitive input. Instead, it relies on the effective integration of multiple brain regions and functional areas, including executive functions, complex attention, and processing speed.^54^ Previous findings from a longitudinal stroke cohort study showed that higher MoCA scores at rehabilitation discharge were associated with higher step counts 2 years later.^18^ Similarly, a study using self-reported physical activity found that higher levels of physical activity were associated with higher MoCA scores after a stroke.^17^ Since the standardized and unstandardized coefficients from our results were both borderline significant, with one estimate slightly below and one slightly above the conventional threshold of *P* < 0.05, we want to interpret this association in our cohort with caution.

We further observed that higher step counts were associated with better performance on complex attention (TMT-A *z*-scores) and executive function (TMT-B *z*-scores) over time. In non-stroke older adults, an accelerometer-based study demonstrated a dose-response relationship between greater engagement in moderate-to-vigorous physical activity and preserved memory and executive function over time.^55^ Supporting this, Galle *et al*.^56^ (2023) reported a cross-sectional association between self-reported physical activity and executive function in older adults that persisted over 16 years. However, they also demonstrated that the level of self-reported physical activity in late adulthood was not related to the rate of decline in executive functions over time. This indicates that individuals with higher physical activity levels at baseline tended to have better executive functions on average, but their rate of decline was similar to those with lower baseline physical activity levels.^56^ Opposed to our hypothesis, we did not observe a between-person association between step count and memory as proxied by the WLMT delayed recall test *z*-scores, possibly due to the largely normative memory performance in our cohort (Table 2). Overall, variations in findings may stem from differences in physical activity measurement tools, cognitive domain assessments, follow-up durations, and study populations across those studies.

### No within-person associations of step count and cognition

In contrast to our hypothesis of a positive feedback loop where better cognitive performance than the expected usual would enable higher engagement in walking, which in turn would further be associated with better cognitive outcomes, we did not find evidence of a bidirectional association between changes in average daily step count after stroke and changes in global cognitive function, memory, executive function, and complex attention in this cohort. At the within-person level, this study did not reveal any dynamic associations between changes from expected post-stroke step count and changes from expected cognitive performance outcomes at any time.

This result seems to contrast with the findings of others. A large cohort study in older adults found a bidirectional positive relationship between physical activity and executive function, despite comparable time lags to our study.^57^ However that study relied on self-reported physical activity and did not separate within- and between-person associations, which may account for the differing results.^57^

Our results also seem to contrast with findings from randomized controlled trials in the chronic phase after stroke, which suggest that physical activity interventions, i.e. changes in physical activity, improved cognitive function.^15^ Threshold effects might explain this, and could have occurred for several reasons. First, changes in a person's average step count and cognitive test scores in this observational study were rather modest. Previous research suggests that changes in physical activity levels only benefit cognition in healthy older adults^58^ and people following stroke when certain thresholds of change are met and interventions are followed consistently.^59^ However, observational studies describe changes in post-stroke physical activity and cognitive trajectories 3 months after the stroke as rather modest.^37,60^ Accordingly, in this study, the intraclass Correlation Coefficients of step count and cognitive scores across measurement occasions were estimated between 0.64 and 0.72. As this value indicates agreement between measurements, it suggests that most of the variance in all these variables was between persons, and less variation was observed at the within-person level. This might also explain the differing results of intervention-based studies, which typically lasted at least 4 weeks for potential effects to accumulate and be detectable.^15^ Second, in the present study, we used step count as a measure of physical activity, considering steps accumulated at any intensity, frequency, and duration, and not accounting for static or resistive physical activities, such as strength training. This may have diluted potential associations. A large accelerometer-based study using Mendelian randomization in the general population found that an increased fraction of both moderate and vigorous physical activity was associated with increased cognitive functioning, whereas average physical activity on cognitive functioning showed no effects.^61^ They also found no evidence of a reverse causal effect from cognitive functioning on any physical activity measures.^61^ Another population-based study linked a higher number of steps with a lower risk of all-cause dementia, also suggesting that higher cadence, thus intensity, would potentially provide additional risk reduction.^62^ In our cohort, all steps were accumulated at light to moderate intensities, with none at high intensities (above 6 METs).

The interpretation of within versus between-person differences is not straightforward. Significant between-person associations can be interpreted as reflecting underlying common factors (e.g. genetic, environmental or personal factors) that predisposed individuals in this cohort to high or low levels in step count and global cognitive function, complex attention, and executive function to co-occur. Yet, it is also possible that processes that link within-person associations between step count and cognition operate on a different timescale, for example, changes occurring before the study or within intervals shorter or longer than the measurement timepoint. Research indicates that too long or short measurement intervals in relation to the process of interest might mask within-person dynamics, resulting in apparent relationships that may solely represent between-person variations.^63^ This explanation is supported by several randomized controlled trials (RCTs) showing that the cognitive benefits of exercise tend to diminish only weeks after an intervention period has ended.^64-66^ In line with this reasoning, few of the autoregressive pathways for step count or cognitive function scores reached significance in our study. In other words, when a person scored above or below their expected level in either of the variables, it was unlikely that similar high or low scores were achieved at subsequent follow-ups. This does not imply that these variables lacked stability over time within individuals; rather, it indicates there were no observable within-person carry-over effects across the observed time interval.^45^ The borderline negative autoregressive pathways for step count in some of the models might be explained by ‘regression to the mean’, a statistical phenomenon describing that unusually large or small measurements tend to be followed by measurements that are closer to the mean.^67^ Future studies should consider implementing shorter time intervals between follow-ups and exploring how long the potential effects of changes in cognitive function and step count on each other persist over time.

Further, engaging in regular physical activity in midlife and maintaining that throughout life has been shown to offer the most long-term benefits for reducing the risk of cognitive decline.^10,13^ This might imply that a longer follow-up might be necessary to be able to capture clinically relevant changes in cognitive function and trajectories. Cognitive benefits from physical activity have been described to stem from the cumulative effects of improved cardiovascular health, improved cerebral blood flow, promotion of neuroplasticity via brain-derived neurotrophic factor pathways, and modulation of neuroinflammation and oxidative stress.^22,68^ Taken together, the insignificant within-person associations across all models showed that temporary deviations from a person’s average level in physical activity or cognition were not predictive of mutual long-term changes.

### Strengths and limitations

This study has several strengths. This study spanned over 3 years, using a three-wave longitudinal design. We conducted a comprehensive cognitive test battery that includes assessments of both global and domain-specific cognitive functions. The use of normalized cognitive scores enabled us to understand how cognitive outcomes compare with what would be expected for similar age, sex and educational levels in a normative sample. Yet, we believe that the overall observed improvements in cognitive function over time could reflect a combination of learning effects and participant attrition. At the same time, the number of participants being normalized against an older age group (Supplementary material on normalization) suggests that, particularly, the *z*-scores of the MoCA and the WLMT delayed recall test overestimated performance, as our participants had a wider age range including younger participants than those in the normative sample. The number of TMT-A and TMT-B tests that were interrupted at the maximum time due to cognitive difficulties further suggests the presence of ceiling effects. The use of an objective measurement tool to capture the average step counts longitudinally in a large group of stroke survivors is another strength. The activPAL accelerometer has been shown to accurately detect steps; however, it has also been found to underestimate steps at slow cadences and during brief walking bouts, which might have contributed to step count underestimations, especially among the frailest participants in our sample.^35,69^ Nevertheless, the mean (SD) mRS score of 1.42 (0.95) at 3 months indicates that this may have affected only few participants.

Despite the obvious advantage of RI-CLPM over cross-sectional designs or longitudinal studies that do not control for the prior level of outcomes or separate between- from within-person variance, this study was not experimental. Causal inference, therefore, requires that there be no unmeasured confounders affecting step count and cognitive outcomes.^45^ This assumption is difficult because risk factors often occur in clusters. While the RI-CLPM accounts for time-invariant unobserved confounding, it cannot control for time-varying sources of unobserved confounding within individuals.^70^ It is possible that known and unknown time-varying factors, such as systemic inflammatory biomarkers, metabolites,^71^ or depressive symptoms^72^ influenced the relationship. Additionally, the time-varying variable of social engagement was not considered in this study. It has been shown that social activity accounted for about 23% of steps taken per day in ambulatory stroke survivors, which exceeded the ∼18% contributed by structured exercise.^73^ Future studies may therefore consider social engagement, as its role in cognition after stroke is not yet fully understood.^74^

The potential for missing data across the different cognitive tests was influenced by the Nor-COAST protocol, which prioritized the MoCA over other assessments when testers were unable to administer the full cognitive test battery. As a result, there was minimal missing data in measures of global cognitive function, whereas most missing data was observed in the delayed recall 10-word test. Although the statistical model addresses missing data, it is important to note that most participants had incomplete data at later follow-ups, which may have led to healthy survivor effects (see Supplementary Table 4 and Supplementary Fig. 1). Additionally, selection bias might have occurred at the time of inclusion. Since cognitive testing and accelerometer provision required in-person meetings, the most vulnerable individuals were excluded. A comparison of included versus excluded participants of the main Nor-COAST study (Supplementary Table 2) revealed that included participants were younger, more educated, experienced milder strokes, and were more independent in daily activities compared with those excluded. Nevertheless, by employing IPW, selection bias and attrition, including death and loss to follow-up from baseline to 3 months, were accounted for. In Supplementary Fig. 3, we show that IPW was effective in balancing covariates. Similar to the majority of the overall Norwegian stroke population, most participants in this study experienced mild strokes.^33^ However, Supplementary Figs 3 and 4 also indicate a tendency to include a higher proportion of mild strokes, even after IPW, and fewer participants with moderate to severe strokes compared with the overall Norwegian stroke population.

Although aphasia was a rare reason for excluding participants in this study (5 people who could not perform the MoCA or the delayed recall 10-word test), difficulties in cognitive evaluation in people with aphasia are common causes of exclusion in clinical studies in general. We note that aphasia and language problems indicate a post-stroke deficit affecting 20% of the Norwegian stroke population^33^; and 16.8% in the overall Nor-COAST sample.

A significant part of cognitive maintenance and preventing cognitive impairment involves preventing new strokes. Future studies should consider including new strokes as time-variant variables in their analyses, as we did not have reliable data on this. Additionally, they should consider more frequent follow-ups to capture the cross-lagged relationship between physical activity and cognitive function over shorter intervals, considering the potential effects of setting (inside or outside/alone or with others),^75^ type, and intensity of physical activity. Home visits for those participants unable to meet in person for testing may prevent the systematic exclusion of the frailest individuals. This insight could guide interventions aimed at maintaining both variables over the long term, likely through multimodal and sustained approaches.

## Conclusion

This study examined the association between average daily step count and both global and domain-specific cognitive functions after stroke. Over time, better performance in complex attention and executive function tended to be observed together with higher levels of step count. Since the findings for global cognitive function and memory were insignificant, we conclude that higher step counts are differentially associated with cognitive domains following a stroke. Further, there was no evidence that, at an individual level, changes in either variable (step count/any cognitive domain) within a person were associated with subsequent changes in the other over time. Our findings suggest there are no bidirectional effects of step count and cognitive performance after stroke across the observed time periods. Therefore, stable, long-lasting differences between individuals, rather than changes that happen within individuals over time, describe the link between step count and cognitive functions.

## Supplementary Material

fcag367_Supplementary_Data

## Data Availability

Following consideration by the Nor-COAST committee, a portion of the data may be available upon request for qualified researchers. Any requests to access the data should be directed to the corresponding author (geske.luzum@ntnu.no). We provide the code used in the Supplementary material.
