# Supplementary material for: Longitudinal associations between physical activity and cognition post-stroke: the Nor-COAST study
Source: Brain Commun. 2026 Oct 12;8(5):fcag367. doi: 10.1093/braincomms/fcag367 (PMC13655615; doi:10.1093/braincomms/fcag367)
Supplement: Supplementary file 1 [file fcag367_supplementary_data.pdf]

Supplementary Material:

Longitudinal associations between physical activity and cognition post-stroke: The Nor-COAST study

Table of Supplementary Content

Normalization of cognitive test scores 2

Table of Supplementary Tables

Supplementary Table 1 Reference data used to normalize cognitive scores in our participants..............3
Supplementary Table 2 A comparison of included versus excluded participants....................................4
Supplementary Table 3 RI-CLPM fit indices 5
Supplementary Table 4 Exploring attrition: Baseline characteristics across timepoints..........................5
Supplementary Table 5 Inverse Probability Weights 7
Supplementary Table 6 Did weighting make the study sample representative of the overall Nor-COAST population? 9
Supplementary Table 7 Within and between-person associations of step-count and cognitive outcomes using a RI-CLPM 12

Table of Supplementary Figures

Supplementary Figure 1 Exploring attrition: Random intercepts by data contribution (N=468)..........6
Supplementary Figure 2 Inverse probability weight (IPW) histogram (N=468)....................................8
Supplementary Figure 3 Covariate balance pre- and post-inverse probability weighting 10
Supplementary Figure 4 Comparing stroke severity at admission against the overall Norwegian stroke population 11

Normalization of cognitive test scores

Z-scores normalized by the mean and standard deviation of the normative data were calculated from the raw scores of the cognitive tests. Lower z-scores indicate poorer outcomes. All normative data were derived from European samples. Both the Montreal Cognitive Assessment (MoCA) and the Word List Memory and Recall Test (WLMT) delayed recall test were normalized using a large population-based Norwegian sample of cognitively healthy older adults aged 70 to 90 years.[1,2] Additionally, for MoCA, we used a normative test data set from Sweden covering individuals outside the age range of 70 to 90.[3] For the WLMT delayed recall test, we added a normative test data set from Germany for individuals aged 60 to 69.[4] Normative data for the Trail Making Test A (TMT-A) and Trail Making Test B (TMT-B) tests were based on a Norwegian sample aged 41 to 84.[5]

This cohort had an age range of 33 to 96 years, and consequently, not all participants fell within the specified age range for the norms. Participants outside the specified age groups were assigned to the nearest age group with normative data. For the MoCA score, 9 people were older than 90 years who contributed data at 3 months (Engedal covers 70 to 90), and 112 people were younger than 65 (Borland covers down to 65: mean: 56.0 (percentiles: 51.5 to 62)). For TMT-A and TMT-B, the number of participants outside the specified age range of 41 to 84 years at 3 months was 59 (12.61%) for TMT-A and 57 (12.18%) for TMT-B, respectively. Six (1.28%) participants with data from both tests were younger, while 53 (11.32%) participants (for TMT-A) and 51 (10.90%) participants (for TMT-B) were older (for both tests, median: 88, 25th to 75th percentiles: 86 to 90). For the WLMT delayed recall test 71 (15.17%) were outside the norms; most of them (n=61, 13.03%) were younger than 60 years (median age: 52 years, 25th to 75th percentiles: 48 to 56) at the 3-month follow-up, while 10 (2.14%) individuals were older than 90 years.

Supplementary Table 1 Reference data used to normalize cognitive scores in our participants

Outcome	Cognitive test	Normative data	Age range
Global cognitive function	Global cognitive function (MoCA)	Engedal et al. 2022 [2]<br>Borland et al 2017 [3]	≥70<br><br><70
Complex attention	Trail Making test A (TMT-A)	Espenes et al. 2020 [5]	everyone
Executive function	Trail Making Test B (TMT-B)	Espenes et al. 2020 [5]	everyone
Memory	WLMT delayed recall test (delayed recall)	Wagle et al. 2023 [1]<br>Luck et al. [4]	≥70<br><br><70

The table shows the normative data used to derive the z-scores of all four cognitive outcomes. Abbreviations: Montreal Cognitive Assessment (MoCA), Word List Memory and Recall Test (WLMT)

Supplementary Table 2 A comparison of included versus excluded participants

Characteristics	Included participants (N=468)	Excluded participants (N=347)
Female, n (%)	193 (41.24)	173 (49.86)
Age, years, mean (SD)	70.9 (11.44)	77.13 (11.18)
Median [25th to 75th percentile]	72 [64 to 79]	79 [71 to 85]
Education, years, mean (SD)	12.85 (3.69)	10.80 (3.47)
Living alone, n (%)	145 (30.98)	153 (44.09)
Institutionalized, n (%)	0	9 (2.56)
Diabetes mellitus, n (%)	93 (19.87)	63 (18.16)
BMI at baseline, mean (SD)	26.23 (4.15)	25.58 (4.28)
NIHSS-1, mean (SD)	2.47 (3.60)	5.18 (6.26)
Median [25th to 75th percentile]	1 (0 to 3)	3 [1 to 7]
Lesion type, n (%)		
Ischemic stroke	427 (91.24)	310 (89.34)
Hemorrhagic stroke	41 (8.76)	37 (10.66)
TOAST, n (%)		
Arteriosclerosis	44 (9.40)	33 (10.96)
Cardioembolism	86 (18.38)	87 (28.90)
Small vessel disease	108 (23.08)	39 (12.96)
Other etiology	11 (2.35)	11 (3.65)
Undetermined	219 (46.79)	131 (43.52)
HADS at 3 months, mean (SD)	7.00 (6.00)	9.06 (7.27)
mRS at baseline, mean (SD)	1.92 (1.21)	2.95 (1.48)
mRS at 3 months, mean (SD)	1.42 (0.95)	2.54 (1.48)

The Table compares the characteristics of Nor-COAST participants (Baseline, N = 815) who were included in this study with those who were excluded. Deviating sample size due to missingness for included participants: BMI (N=449); mRS at baseline (N=466); mRS at 3 months (N=463); TOAST (N=412); HADS (N=410) Deviating sample size due to missingness in excluded Nor-COAST participants: BMI (N=294); mRS at baseline (N=345); mRS at 3 months (N=238); TOAST (N=301); HADS-D (N=142)

Abbreviations: Body Mass Index (BMI), National Institutes of Health Stroke Scale measured 1 day after hospital admission (NIHSS-1), Categorization of subtypes of ischemic stroke developed for the Trial of Org 10172 in Acute Stroke Treatment (TOAST), modified Rankin Scale (mRS)

Supplementary Table 3 RI-CLPM fit indices

Fit indices	MoCA	TMT-A	TMT-B	Delayed recall
Model Chi-Square	36.57	46.85	30.96	23.98
Degrees of freedom	21	21	21	21
p-value (Chi-square)	0.02	<0.01	0.07	0.29
CFI	0.99	0.98	0.99	0.99
RMSEA (95% CI)	0.04 (0.02 to 0.06)	0.05 (0.03 to 0.07)	0.03 (0.00 to 0.05)	0.02 (<0.01 to 0.04)
SRMR	0.03	0.033	0.03	0.02
PNFI	0.45	0.45	0.45	0.46

Fit indices of the final adjusted weighted RI-CLPMs are presented as suggested by the Guidelines for Determining Model Fit in structural equation modelling.[6] In accordance, we present and review fit indices for each model (N=468), but we did not let model fit alone drive decisions on modelling procedure. Abbreviations: Random Intercept Cross-Lagged Panel Model (RI-CLPM); Montreal Cognitive Assessment (MoCA); Trail Making Test A and B (TMT-A, TMT-B); Comparative fit index (CFI); Root mean square error of approximation (RMSEA); standardized root mean square residual (SRMR); Parsimonious Normed Fit Index (PNFI)

Supplementary Table 4 Exploring attrition: Baseline characteristics across timepoints

	Included participants contributing step and cognitive data at		
**Baseline characteristics**	**3 months (N=460)**	**18 months (N=405)**	**36 months (N=278)**
Female, n (%)	192 (41.7)	168 (41.5)	114 (41.0)
Age, yrs, mean (SD)	70.9 (11.4)	70.3 (11.2)	68.8 (11.2)
Median [25th to 75th percentile]	72 [64 to 79]	72 [63 to 79]	70 [62 to 77]
Education, yrs., mean (SD)	12.8 (3.7)	13.0 (3.7)	13.3 (3.7)
Living alone, n (%)	143 (31.1)	119 (29.4)	78 (28.1)
NIHSS-1, mean (SD)	2.5 (3.6)	2.5 (3.7)	2.4 (3.9)
Median [25th to 75th percentile]	1 [0 to 3]	1 [0 to 3]	1 [0 to 3]
Lesion type, n (%)			
Ischemic stroke	419 (91.1)	372 (91.9)	254 (91.4)
Hemorrhagic stroke	41 (8.9)	33 (8.1)	24 (8.6)
mRS* at baseline, mean (SD)	1.93 (1.22)	1.87 (1.21)	1.75 (1.19)

The table displays the baseline characteristics of participants included (N=468) across all three measurement time points, only for those who contributed any data at each specific time point (accelerometer data or data from MoCA, TMT-A, TMT-B, delayed recall 10-word test) during follow-ups. Abbreviations: National Institutes of Health Stroke Scale measured 1 day after hospital admission (NIHSS-1); Modified Rankin Scale (mRS); *mRS at baseline: 3 months: N=458, 18 months: N=403; 36 months: N=276.

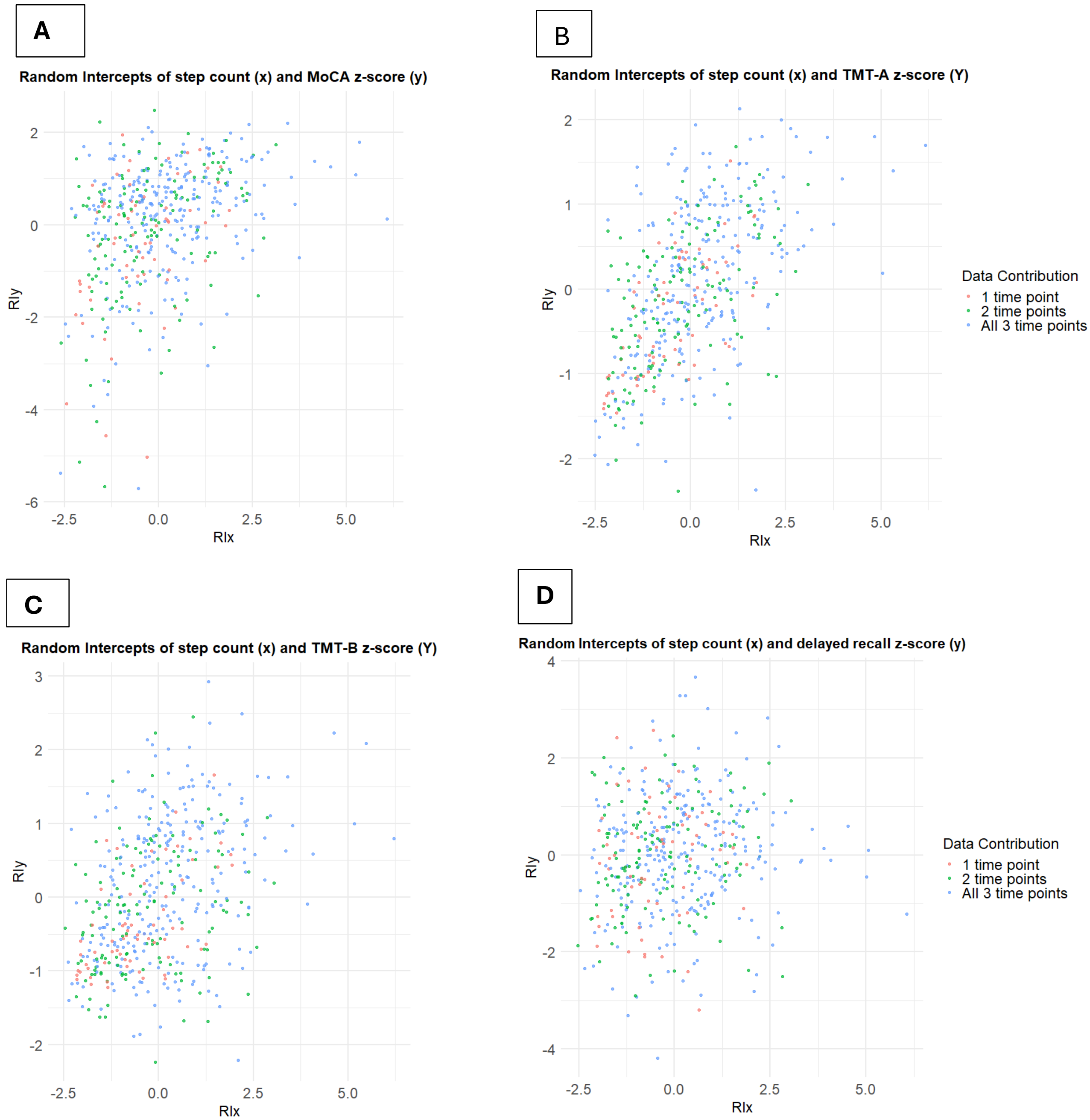

Supplementary Figure 1 Exploring attrition: Random intercepts by data contribution (N=468)

In this Figure, we visually explored whether those who contributed more data to the study had higher random intercepts, thereby suggesting that they are, on average, performing better, and that their continued participation might have been selective. We plotted the random intercepts (RI) of all four Random Intercept Cross-lagged Panel models (RI-CLPMs) against each other. The RIs of step count were plotted against the RIs of the z-scores of Montreal Cognitive Assessment (MoCA) in Graph A, Trail Making Test A (TMT-A) in Graph B, Trail Making Test B (TMT-B) in Graph C, and the delayed recall test in Graph D. RIx represents the random intercept of step count (scaled by a factor of 1/1000); RIy represents the RI of the cognitive z-scores, respectively. The zero for both axes equals the grand mean. The individual RIs are centered around the grand mean in different colors. The colours indicate the maximum data contribution of either of the variables. Dots below zero indicate below-average; above zero indicate above-average values. While there may be a slight tendency for individuals with more data contribution (blue) to have overall higher RIs, from these plots, we conclude an overall good distribution of individuals with differing data contribution (different colors) around the grand means.

Supplementary Table 5 Inverse Probability Weights

Years of education	Age	mRS	Trimmed IPW
<11 years	<70 years	mRS <3	1.46
11-13 years	<70 years	mRS <3	1.25
13+ years	<70 years	mRS <3	1.18
<11 years	<70 years	mRS 3+	2.75
11-13 years	<70 years	mRS 3+	1.97
13+ years	<70 years	mRS 3+	1.71
<11 years	70-79 years	mRS <3	1.52
11-13 years	70-79 years	mRS <3	1.29
13+ years	70-79 years	mRS <3	1.21
<11 years	70-79 years	mRS 3+	3.00
11-13 years	70-79 years	mRS 3+	2.11
13+ years	70-79 years	mRS 3+	1.81
<11 years	80+ years	mRS <3	1.87
11-13 years	80+ years	mRS <3	1.48
13+ years	80+ years	mRS <3	1.35
<11 years	80+ years	mRS 3+	3.00
11-13 years	80+ years	mRS 3+	2.85
13+ years	80+ years	mRS 3+	2.35

To balance baseline characteristics of included and excluded participants in Nor-COAST and account for selection bias, we employed a logistic regression model. A dichotomous variable indicating whether participants were included and excluded was set as the dependent variable. Age, education, and the modified Rankin Scale (mRS) as a global measure of independence in daily activities were used as independent variables. Weights were calculated for each included individual as 1/propensity score. Weights were trimmed at the 97th percentile, resulting in 14 participants (education group: <11 years, age group: 80+ years, mRS: 3+) being assigned somewhat lower weights (IPW: 4.36 to 3.00). A sensitivity analysis, including trimmed versus untrimmed weights in the main models, showed similar results. Individuals with the lowest education, the highest dependence in daily living, and the oldest age group at baseline were assigned the highest weights. As a result, these individuals' relative influence on the study's results was about three times higher than the least weighted group (IPW: 1.18), which includes individuals with the highest years of education, the youngest age group, and the least ADL dependence. A histogram of the trimmed IPW variable is displayed below. Abbreviations: modified Rankin Scale (mRS); Inverse Probability Weights (IPW).

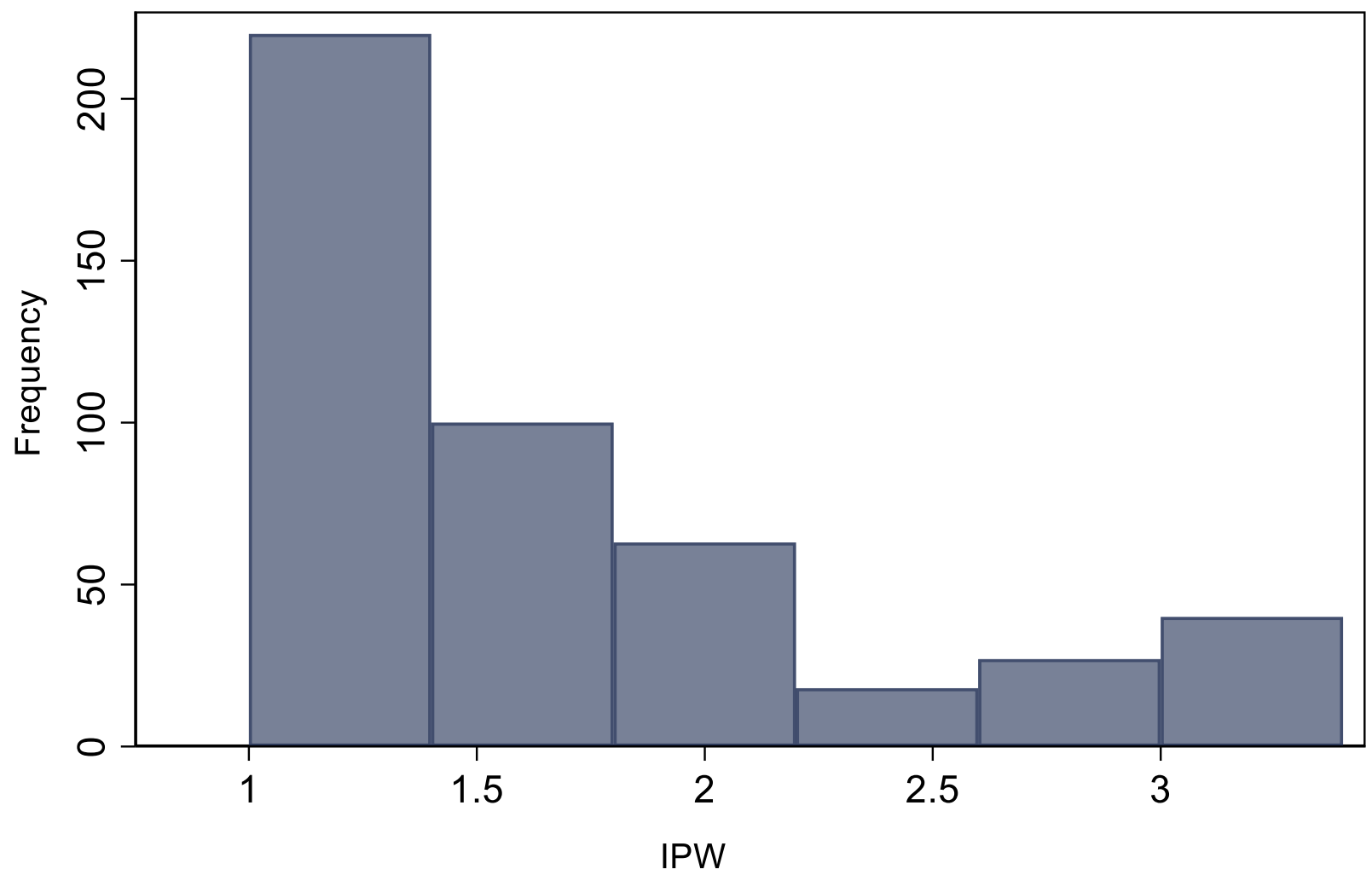

Supplementary Figure 2 Inverse probability weight (IPW) histogram (N=468)

Visualization of the trimmed IPWs using a histogram (also see Supplementary Table 5).

Supplementary Table 6 Did weighting make the study sample representative of the overall Nor-COAST population?

Characteristics	Overall Nor-COAST sample (N=815)	Participants pre-weighting (N=468)	SMD	Participants post-weighting (N=468)	SMD
Female, n (%)	366 (44.91)	193 (41.24)	0.07	208 (44.48)	0.01
Age, yrs, mean (SD)	73.53 (11.74)	70.86 (11.44)	0.23	72.52 (11.16)	0.09
Education, yrs., mean (SD)	11.97 (3.73)	12.85 (3.69)	-0.24	12.28 (3.66)	-0.08
Living alone, n (%)	298 (36.56)	145 (30.98)	0.12	163 (34.87)	0.04
Diabetes mellitus, n (%)	156 (19.14)	93 (19.87)	0.02	94 (20.17)	-0.02
NIHSS-1, mean (SD)	3.58 (5.04)	2.47 (3.60)	0.25	2.96 (3.90)	0.14
BMI	25.97 (4.21)	26.23 (4.15)	-0.07	26.15 (4.20)	-0.04
Lesion type, n (%)			0.03		<0.01
Ischemic stroke	737 (90.43)	427 (91.24)		423 (90.40)	
Hemorrhagic stroke	78 (9.57)	41 (8.76)		44 (9.60)	
mRS, baseline, mean (SD)	2.36 (1.43)	1.93 (1.21)	0.33	2.24 (1.28)	0.09

The Table displays the Standardized Mean Difference (SMD) of the baseline characteristics before and after inverse probability weighting (IPW) of this study sample compared to the overall (unweighted) Nor-COAST study sample to evaluate whether IPW was successful. As a rule of thumb, an absolute standardized difference of 0.10 or more indicates that the characteristic of interest is imbalanced between groups[7], implying a selection bias. Abbreviations: National Institutes of Health Stroke Scale measured 1 day after hospital admission (NIHSS-1), Body Mass Index (BMI), modified Rankin Scale (mRS); Deviating sample size: NIHSS-1: N=792; mRS: N=811 in the overall Nor-COAST sample and N=466 in the study sample

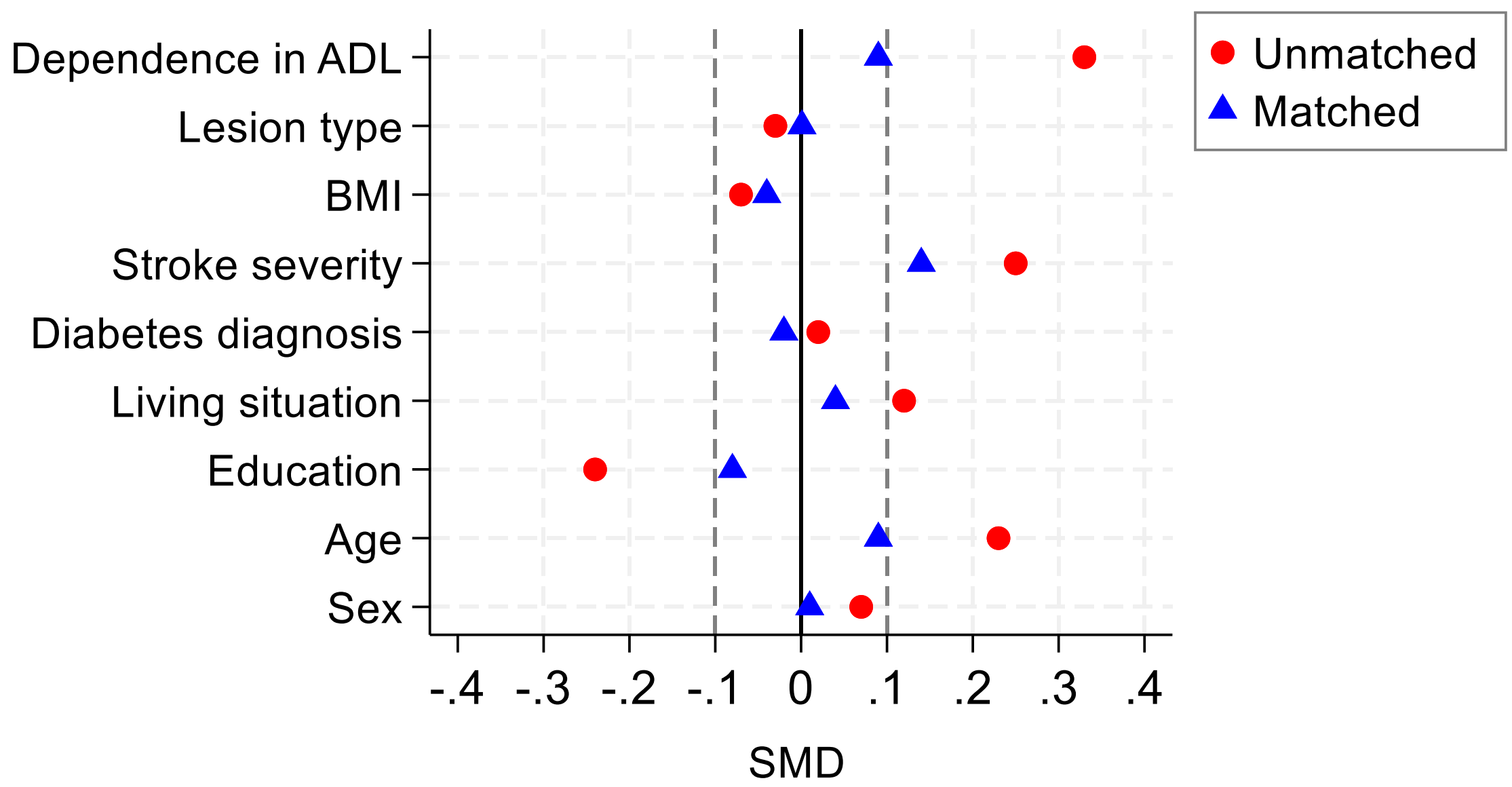

Supplementary Figure 3 Covariate balance pre- and post-inverse probability weighting

This Figure visualizes the covariate balance comparing our study sample (N = 468) to the total Nor-COAST cohort (N = 815) based on the findings displayed in Supplementary Table 6. We present the Standardized Mean Differences (SMDs) for baseline characteristics before (unmatched) and after (matched) inverse probability weighting (IPW). As a general guideline, an absolute standardized mean difference (SMD) ≤0.10 indicates balanced characteristics between groups.[7] The Figure shows that balance improved on almost all variables after weighting, resulting in all but one falling below that threshold. The SMD of 0.14 for dependence in stroke severity (NIHSS-1) suggests a remaining imbalance of baseline characteristics between the overall Nor-COAST sample and this study sample, even after weighting. Yet, IPW was successful for all other characteristics. A comparison of weighted stroke severity in this sample versus the overall Nor-COAST sample and the overall Norwegian stroke population is illustrated in Supplementary Figure 4. Abbreviations: Body Mass Index (BMI), National Institutes of Health Stroke Scale measured 1 day after hospital admission (NIHSS-1).

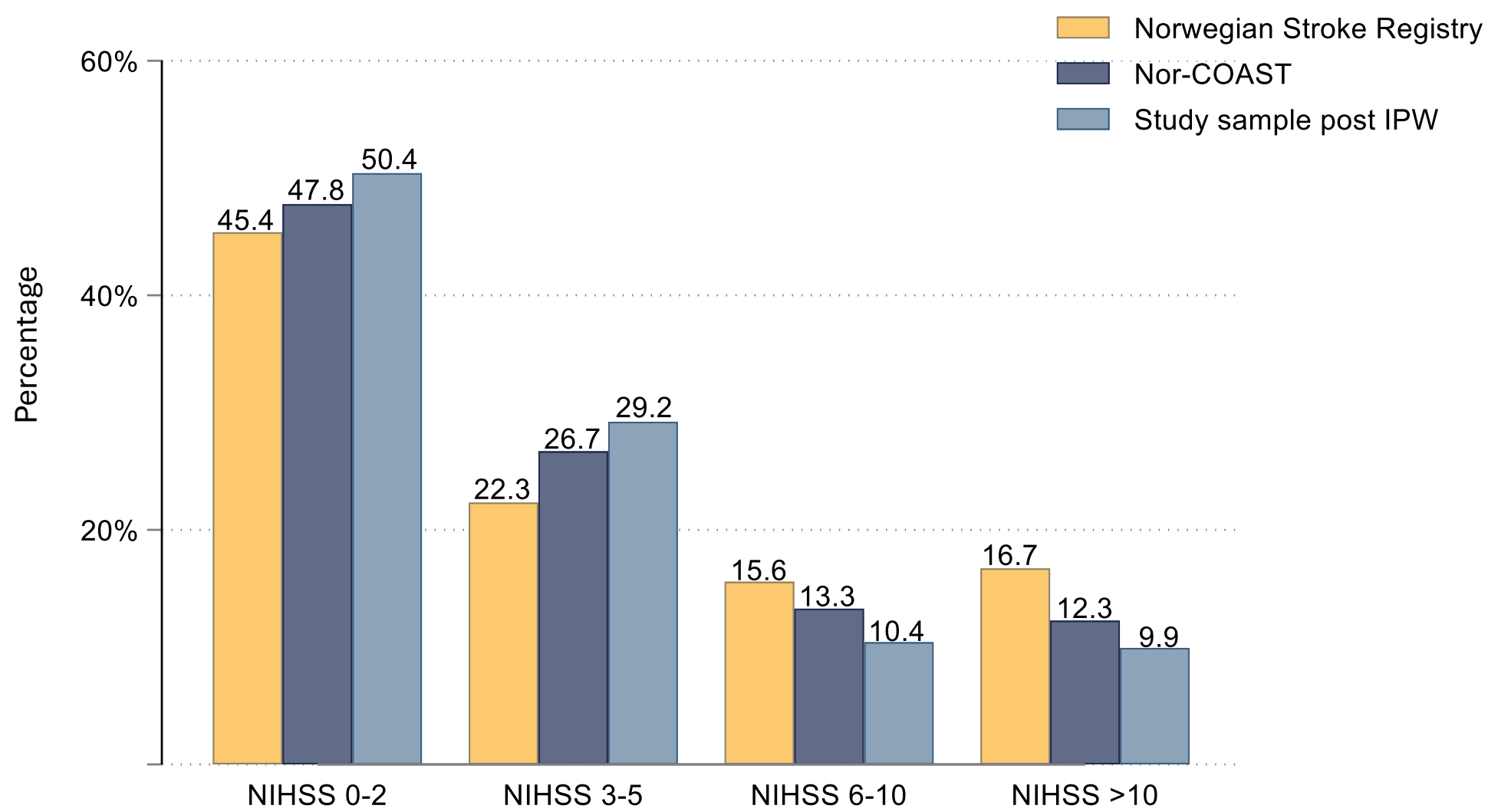

Supplementary Figure 4 Comparing stroke severity at admission against the overall Norwegian stroke population

The figure is a simple bar graph to visually compare stroke severity in our study sample (N = 468) before inverse probability weighting (IPW) against the overall Nor-COAST sample (N=815) and all individuals registered with a new stroke (N = 8972) who were assessed with the National Institutes of Health Stroke Scale (NIHSS-0) at hospital admission (88.3%) in Norway in 2023 as registered in the Norwegian Stroke Registry. The Norwegian stroke registry defines mild strokes as those with an NIHSS score of 0 to 5.[8] Similar to the majority of the overall Norwegian stroke population (shown in yellow), most participants in this study (light blue) experienced mild strokes. However, the figure also indicates a tendency to include a higher proportion of mild strokes, even after IPW, and fewer participants with moderate to severe strokes. Note: To compare with stroke registry data, we present the NIHSS-0 at hospital admission in this graph; otherwise, we report the NIHSS-1 assessed on day 1 after admission.

Supplementary Table 7 Within and between-person associations of step-count and cognitive outcomes using a RI-CLPM

		Unstandardized			Standardized			
	**Parameter**	**B**	**SE**	**p**	**β**	**p**	**95% CI**	
				**MoCA z-score**				
Within-person	Autoregressive pathways							
	alpha2	-0.30	0.15	0.05	-0.60	0.07	-1.23	0.04
	alpha3	-0.41	0.36	0.25	-0.20	0.19	-0.50	0.10
	delta2	0.02	0.35	0.97	0.01	0.97	-0.62	0.65
	delta3	0.35	0.17	0.04	0.32	0.06	-0.01	0.65
	Cross-lagged pathways							
	beta2	-0.13	0.16	0.42	-0.30	0.38	-0.96	0.37
	beta3	0.17	0.14	0.25	0.20	0.27	-0.16	0.55
	gamma2	-0.08	0.23	0.74	-0.06	0.75	-0.45	0.32
	gamma3	0.26	0.27	0.34	0.10	0.37	-0.11	0.31
	Cross-sectional pathways							
	T1	-0.11	0.14	0.44	-0.12	0.46	-0.44	0.20
	T2	0.03	0.20	0.87	0.09	0.86	-0.86	1.04
	T3	0.16	0.13	0.21	0.16	0.22	-0.20	0.40
Between-person	RI (kappa-omega)	0.25	0.14	0.06	0.14	0.04	0.01	0.28
				**TMT-A z-score**				
Within-person	Autoregressive pathways							
	alpha2	-0.30	0.15	0.04	-0.59	0.07	-1.24	0.06
	alpha3	-0.45	0.38	0.24	-0.22	0.18	-0.54	0.10
	delta2	0.19	0.15	0.21	0.20	0.20	-0.11	0.51
	delta3	0.07	0.20	0.72	0.07	0.72	-0.31	0.45
	Cross-lagged pathways							
	beta2	-0.11	0.13	0.37	-0.21	0.42	-0.70	0.29
	beta3	0.11	0.20	0.59	0.09	0.59	-0.24	0.42
	gamma2	-0.10	0.11	0.34	-0.12	0.35	-0.36	0.13
	gamma3	0.23	0.24	0.34	0.13	0.36	-0.15	0.40
	Cross-sectional pathways							
	T1	-0.13	0.08	0.08	-0.18	0.08	-0.39	0.02
	T2	0.04	0.09	0.70	0.13	0.68	-0.48	0.74
	T3	0.07	0.09	0.41	0.10	0.43	-0.14	0.34
Between-person	RI (kappa-omega)	**0.24**	**0.08**	**0.001**	**0.24**	**0.002**	**0.09**	**0.40**
				**TMT-B z-score**				
Within-person	Autoregressive pathways							
	alpha2	-0.31	0.18	0.09	-0.59	0.14	-1.37	0.20
	alpha3	-0.31	0.32	0.34	-0.16	0.30	-0.45	0.14
	delta2	0.17	0.13	0.20	0.18	0.18	-0.08	0.44
	delta3	-0.10	0.16	0.54	-0.10	0.53	-0.40	0.21
	Cross-lagged pathways							
	beta2	-0.11	0.16	0.51	-0.18	0.53	-0.75	0.38
	beta3	0.34	0.19	0.07	0.27	0.07	-0.02	0.56
	gamma2	-0.03	0.11	0.76	-0.04	0.76	-0.29	0.21
	gamma3	0.13	0.26	0.63	0.08	0.65	-0.26	0.41

	Cross-sectional pathways							
	T1	-0.08	0.09	0.33	-0.12	0.34	-0.38	0.13
	T2	-0.01	0.09	0.93	-0.03	0.93	-0.74	0.68
	T3	0.16	0.09	0.07	0.25	0.11	-0.06	0.55
Between-person	RI (kappa-omega)	**0.24**	**0.08**	**0.002**	**0.22**	**0.001**	**0.09**	**0.34**
		**WLMT delayed recall test z-score**						
Within-person	Autoregressive pathways							
	alpha2	-0.26	0.13	0.04	-0.51	0.07	-1.05	0.04
	alpha3	-0.31	0.35	0.38	-0.15	0.33	-0.46	0.16
	delta2	0.17	0.17	0.31	0.15	0.31	-0.15	0.45
	delta3	0.15	0.14	0.27	0.17	0.27	-0.13	0.46
	Cross-lagged pathways							
	beta2	0.08	0.11	0.48	0.16	0.48	-0.28	0.60
	beta3	-0.20	0.13	0.13	-0.22	0.11	-0.50	0.05
	gamma2	-0.21	0.15	0.16	-0.18	0.15	-0.42	0.06
	gamma3	0.36	0.28	0.19	0.18	0.20	-0.09	0.44
	Cross-sectional pathways							
	T1	0.01	0.10	0.91	0.01	0.91	-0.21	0.24
	T2	-0.10	0.13	0.46	-0.23	0.48	-0.88	0.41
	T3	-0.001	0.12	0.99	-0.002	0.99	-0.28	0.28
Between-person	RI (kappa-omega)	0.13	0.09	0.18	0.09	0.18	-0.04	0.22

The Table displays the output of all 4 Random Intercept Cross-lagged Panel Models (RI-CLPM) with unstandardized and standardized estimates. A one-unit change in the unstandardized coefficients of cognitive z-scores corresponds to one standard deviation. A one-unit change in the unstandardized coefficients of step count corresponds to 1000 steps/day. Estimates that remained significant after Holm-Bonferroni correction are presented in bold. Abbreviations: Random Intercept (RI), Timepoint of follow-up (T1 = 3 months, T2 = 18 months, T3 = 36 months), Montreal Cognitive Assessment (MoCA), Trail Making Test A (TMT-A), Trail Making Test B (TMT-B), Word List Memory and Recall Test (WLMT)

Code in Stata

This is the code used to generate z-scores and IPW in the Nor-COAST data set using stata:

```
* Create the summary scores of MoCA across all 4 timepoints

gen MoCA_T0 = MoCA_1_T0 + MoCA_2_T0 + MoCA_3_T0 + MoCA_4_T0 +MoCA_5_T0 +MoCA_6_T0 +MoCA_7_T0
+MoCA_8_T0 +MoCA_9_T0 +MoCA_10_T0+ MoCA_11_T0+ MoCA_12_T0+ MoCA_13_T0 +MoCA_14_T0
+MoCA_15_T0 +MoCA_16_T0 +MoCA_17_T0 +MoCA_18_T0 +MoCA_19_T0 +MoCA_20_T0 +MoCA_21_T0
+MoCA_22_T0 +MoCA_23_T0 + MoCA_24_T0+ MoCA_25_T0+ MoCA_26_T0 +MoCA_27_T0 +MoCA_28_T0

replace MoCA_T0 = MoCA_T0 + 1 if education_year_T0 <12

sum MoCA_T0

hist MoCA_T0

sum MoCA_T0 if MoCA_T0 <26

sum MoCA_T0 if MoCA_T0 >25 & MoCA_T0 <31

gen MoCA_T1 = MoCA_1_T1 + MoCA_2_T1 + MoCA_3_T1 + MoCA_4_T1 +MoCA_5_T1 +MoCA_6_T1 +MoCA_7_T1
+MoCA_8_T1 +MoCA_9_T1 +MoCA_10_T1+ MoCA_11_T1+ MoCA_12_T1+ MoCA_13_T1 +MoCA_14_T1
+MoCA_15_T1 +MoCA_16_T1 +MoCA_17_T1 +MoCA_18_T1 +MoCA_19_T1 +MoCA_20_T1 +MoCA_21_T1
+MoCA_22_T1 +MoCA_23_T1 + MoCA_24_T1+ MoCA_25_T1+ MoCA_26_T1 +MoCA_27_T1 +MoCA_28_T1

replace MoCA_T1 = MoCA_T1 + 1 if education_year_T0 <13   // only do that if i dont use the z-scores!

replace MoCA_T1 =30 if MoCA_T1 ==31

sum MoCA_T1

hist MoCA_T1

sum MoCA_T1 if MoCA_T1 <26

sum MoCA_T1 if MoCA_T1 >25 & MoCA_T1 <31


gen MoCA_T2= MoCA_1_T2 + MoCA_2_T2 + MoCA_3_T2 + MoCA_4_T2 +MoCA_5_T2 +MoCA_6_T2 +MoCA_7_T2
+MoCA_8_T2 +MoCA_9_T2 +MoCA_10_T2+ MoCA_11_T2+ MoCA_12_T2+ MoCA_13_T2 +MoCA_14_T2
+MoCA_15_T2 +MoCA_16_T2 +MoCA_17_T2 +MoCA_18_T2 +MoCA_19_T2 + MoCA_20_T2+ MoCA_21_T2
+MoCA_22_T2 +MoCA_23_T2 +MoCA_24_T2+ MoCA_25_T2+ MoCA_26_T2 +MoCA_27_T2 +MoCA_28_T2

replace MoCA_T2 = MoCA_T2 + 1 if education_year_T0 <13  // only do that if oi dont use the z-scores!

replace MoCA_T2=30 if MoCA_T2==31

sum MoCA_T2

hist MoCA_T2

sum MoCA_T2 if MoCA_T2 <26

sum MoCA_T2 if MoCA_T2 >25 & MoCA_T2 <31


gen MoCA_T3 = MoCA_1_T3 + MoCA_2_T3 + MoCA_3_T3 + MoCA_4_T3 +MoCA_5_T3 +MoCA_6_T3 +MoCA_7_T3
+MoCA_8_T3 +MoCA_9_T3 +MoCA_10_T3+ MoCA_11_T3+ MoCA_12_T3+ MoCA_13_T3 +MoCA_14_T3
+MoCA_15_T3 +MoCA_16_T3 +MoCA_17_T3 +MoCA_18_T3 +MoCA_19_T3 +MoCA_20_T3 +MoCA_21_T3
+MoCA_22_T3 +MoCA_23_T3 +MoCA_24_T3+ MoCA_25_T3+ MoCA_26_T3 +MoCA_27_T3 +MoCA_28_T3

replace MoCA_T3 = MoCA_T3 + 1 if education_year_T0 <13  // only do that if not using z scores!

replace MoCA_T3 =30 if MoCA_T3 ==31
```

```
sum MoCA_T3

hist MoCA_T3

sum MoCA_T3 if MoCA_T3 <26

sum MoCA_T3 if MoCA_T3 >25 & MoCA_T3 <31


**OBS: moca is not education adjusted with one point more for people who have 12 or less years of education since we will use normalization data


**# MoCA Normalization #1

*Now generate the Z scores based on aldring og helse, Engedal 2022 HUNT, https://journals.sagepub.com/doi/10.3233/JAD-215442?url_ver=Z39.88-2003&rfr_id=ori:rid:crossref.org&rfr_dat=cr_pub%20%200pubmed


*Generate the age groups according to Engedal,  everyone younger than 70 is assigned to age 70-74 and everyone lder than 90 is assigned to 85+

generate Age_Tbase =((admission_date_T0) - birthdate ) / 365.25   //age at admission

generate Age_T1_detail =((test_date_T1) - birthdate ) / 365.25   //age at 3 months testing

generate Age_T2_detail = ((test_date_T2) - birthdate ) / 365.25  //age at 18 months testing

generate Age_T3_detail = ((test_date_T3) - birthdate ) / 365.25  //age at 36 months testing

gen Age_T1 = round(Age_T1_detail)

gen Age_T2 = round(Age_T2_detail)

gen Age_T3 = round(Age_T3_detail)


gen agegroupT1_Engedal = "<75 years" if Age_T1 <75

replace agegroupT1_Engedal = "75-79 years" if Age_T1 >74 & Age_T1<80

replace agegroupT1_Engedal = "80-84 years" if Age_T1 >79 & Age_T1<85

replace agegroupT1_Engedal = "85+ years" if Age_T1 >84 & Age_T1 !=.

tab agegroupT1_Engedal  //agegroup T1


gen agegroupT2_Engedal = "<75 years" if Age_T2 <75

replace agegroupT2_Engedal = "75-79 years" if Age_T2 >74 & Age_T2<80

replace agegroupT2_Engedal = "80-84 years" if Age_T2 >79 & Age_T2<85

replace agegroupT2_Engedal = "85+ years" if Age_T2 >84 & Age_T2 !=.

tab agegroupT2_Engedal  //agegroup T2


gen agegroupT3_Engedal = "<75 years" if Age_T3 <75

replace agegroupT3_Engedal = "75-79 years" if Age_T3 >74 & Age_T3<80

replace agegroupT3_Engedal = "80-84 years" if Age_T3 >79 & Age_T3<85
```

```
replace agegroupT3_Engedal = "85+ years" if Age_T3 >84 & Age_T3 !=.

tab agegroupT3_Engedal  //ageggroup T3


*Generate the education groups according to Engedal

gen edu_group_engedal = "Compulsary" if education_year_T0 < 11

replace edu_group_engedal = "Secondary" if education_year_T0 >10 & education_year_T0 <14

replace edu_group_engedal = "Tertiary" if education_year_T0 >13 & education_year_T0 !=.

tab edu_group_engedal


******T1*************Generate z-scores MOCA according to Engdedal based on sex, age, education T1

gen z_score_MoCA_T1 = ((MoCA_T1 - 25.1)/2.11) if agegroupT1_Engedal == "<75 years" & edu_group_engedal == "Compulsary" & gender_T0==1  //gender 1 is female, 2 is male

replace z_score_MoCA_T1 =((MoCA_T1 - 24.3)/1.76) if agegroupT1_Engedal == "<75 years" & edu_group_engedal == "Compulsary" & gender_T0==2

replace z_score_MoCA_T1 = ((MoCA_T1 - 24.8)/2.22) if agegroupT1_Engedal == "75-79 years" & edu_group_engedal == "Compulsary" & gender_T0==1

replace z_score_MoCA_T1 =((MoCA_T1 - 24.4)/2.23) if agegroupT1_Engedal == "75-79 years" & edu_group_engedal == "Compulsary" & gender_T0==2

replace z_score_MoCA_T1 = ((MoCA_T1 - 24.2)/2.65) if agegroupT1_Engedal == "80-84 years" & edu_group_engedal == "Compulsary" & gender_T0==1

replace z_score_MoCA_T1 =((MoCA_T1 - 23.7)/2.28) if agegroupT1_Engedal == "80-84 years" & edu_group_engedal == "Compulsary" & gender_T0==2

replace z_score_MoCA_T1 = ((MoCA_T1 - 23.3)/2.07) if agegroupT1_Engedal == "85+ years" & edu_group_engedal == "Compulsary" & gender_T0==1

replace z_score_MoCA_T1 =((MoCA_T1 - 22.9)/2.14) if agegroupT1_Engedal == "85+ years" & edu_group_engedal == "Compulsary" & gender_T0==2   //all women and men of all age groups for COMPULSARY education


replace z_score_MoCA_T1 = ((MoCA_T1 - 25.9)/2.05) if agegroupT1_Engedal == "<75 years" & edu_group_engedal == "Secondary" & gender_T0==1

replace z_score_MoCA_T1 = ((MoCA_T1 - 25.3)/2.08) if agegroupT1_Engedal == "<75 years" & edu_group_engedal == "Secondary" & gender_T0==2

replace z_score_MoCA_T1 = ((MoCA_T1 - 25.4)/2.28) if agegroupT1_Engedal == "75-79 years" & edu_group_engedal == "Secondary" & gender_T0==1

replace z_score_MoCA_T1 =((MoCA_T1 - 25.0)/2.18) if agegroupT1_Engedal == "75-79 years" & edu_group_engedal == "Secondary" & gender_T0==2

replace z_score_MoCA_T1 = ((MoCA_T1 - 25.0)/2.26) if agegroupT1_Engedal == "80-84 years" & edu_group_engedal == "Secondary" & gender_T0==1

replace z_score_MoCA_T1 =((MoCA_T1 - 24.3)/2.13) if agegroupT1_Engedal == "80-84 years" & edu_group_engedal == "Secondary" & gender_T0==2

replace z_score_MoCA_T1 = ((MoCA_T1 - 23.7)/2.41) if agegroupT1_Engedal == "85+ years" & edu_group_engedal == "Secondary" & gender_T0==1
```

```
replace z_score_MoCA_T1 =((MoCA_T1 - 22.9)/1.96) if agegroupT1_Engedal == "85+ years" & edu_group_engedal == "Secondary" & gender_T0==2   //all women and men of all age groups for SECONDARY education


replace z_score_MoCA_T1 = ((MoCA_T1 - 26.9)/1.93) if agegroupT1_Engedal == "<75 years" & edu_group_engedal == "Tertiary" & gender_T0==1

replace z_score_MoCA_T1 =((MoCA_T1 - 26.5)/1.73) if agegroupT1_Engedal == "<75 years" & edu_group_engedal == "Tertiary" & gender_T0==2

replace z_score_MoCA_T1 = ((MoCA_T1 - 26.7)/2.00) if agegroupT1_Engedal == "75-79 years" & edu_group_engedal == "Tertiary" & gender_T0==1

replace z_score_MoCA_T1 =((MoCA_T1 - 26.5)/1.77) if agegroupT1_Engedal == "75-79 years" & edu_group_engedal == "Tertiary" & gender_T0==2

replace z_score_MoCA_T1 = ((MoCA_T1 - 26.1)/2.16) if agegroupT1_Engedal == "80-84 years" & edu_group_engedal == "Tertiary" & gender_T0==1

replace z_score_MoCA_T1 =((MoCA_T1 - 25.3)/2.12) if agegroupT1_Engedal == "80-84 years" & edu_group_engedal == "Tertiary" & gender_T0==2

replace z_score_MoCA_T1 = ((MoCA_T1 - 24.7)/2.34) if agegroupT1_Engedal == "85+ years" & edu_group_engedal == "Tertiary" & gender_T0==1

replace z_score_MoCA_T1 =((MoCA_T1 - 24.8)/1.97) if agegroupT1_Engedal == "85+ years" & edu_group_engedal == "Tertiary" & gender_T0==2   //all women and men of all age groups for TERTIARY education


*adding the z-scores from previous dataset based on Borland (65)for everyone outside the age range 70 to 90

replace z_score_MoCA_T1 = z_score_moca_st_T1 if Age_T1 <70 & z_score_MoCA_T1 !=.

sum z_score_MoCA_T1, detail


*********T2*******************Generate z-scores MOCA according to Engdedal based on sex, age, education T2

gen z_score_MoCA_T2 = ((MoCA_T2 - 25.1)/2.11) if agegroupT2_Engedal == "<75 years" & edu_group_engedal == "Compulsary" & gender_T0==1  //gender 1 is female, 2 is male

replace z_score_MoCA_T2 =((MoCA_T2 - 24.3)/1.76) if agegroupT2_Engedal == "<75 years" & edu_group_engedal == "Compulsary" & gender_T0==2

replace z_score_MoCA_T2 = ((MoCA_T2 - 24.8)/2.22) if agegroupT2_Engedal == "75-79 years" & edu_group_engedal == "Compulsary" & gender_T0==1

replace z_score_MoCA_T2 =((MoCA_T2 - 24.4)/2.23) if agegroupT2_Engedal == "75-79 years" & edu_group_engedal == "Compulsary" & gender_T0==2

replace z_score_MoCA_T2 = ((MoCA_T2 - 24.2)/2.65) if agegroupT2_Engedal == "80-84 years" & edu_group_engedal == "Compulsary" & gender_T0==1

replace z_score_MoCA_T2 =((MoCA_T2 - 23.7)/2.28) if agegroupT2_Engedal == "80-84 years" & edu_group_engedal == "Compulsary" & gender_T0==2

replace z_score_MoCA_T2 = ((MoCA_T2 - 23.3)/2.07) if agegroupT2_Engedal == "85+ years" & edu_group_engedal == "Compulsary" & gender_T0==1

replace z_score_MoCA_T2 =((MoCA_T2 - 22.9)/2.14) if agegroupT2_Engedal == "85+ years" & edu_group_engedal == "Compulsary" & gender_T0==2   //all women and men of all age groups for COMPULSARY education
```

replace z_score_MoCA_T2 = ((MoCA_T2 - 25.9)/2.05) if agegroupT2_Engedal == "<75 years" & edu_group_engedal == "Secondary" & gender_T0==1

replace z_score_MoCA_T2 = ((MoCA_T2 - 25.3)/2.08) if agegroupT2_Engedal == "<75 years" & edu_group_engedal == "Secondary" & gender_T0==2

replace z_score_MoCA_T2 = ((MoCA_T2 - 25.4)/2.28) if agegroupT2_Engedal == "75-79 years" & edu_group_engedal == "Secondary" & gender_T0==1

replace z_score_MoCA_T2 =((MoCA_T2 - 25.0)/2.18) if agegroupT2_Engedal == "75-79 years" & edu_group_engedal == "Secondary" & gender_T0==2

replace z_score_MoCA_T2 = ((MoCA_T2 - 25.0)/2.26) if agegroupT2_Engedal == "80-84 years" & edu_group_engedal == "Secondary" & gender_T0==1

replace z_score_MoCA_T2 =((MoCA_T2 - 24.3)/2.13) if agegroupT2_Engedal == "80-84 years" & edu_group_engedal == "Secondary" & gender_T0==2

replace z_score_MoCA_T2 = ((MoCA_T2 - 23.7)/2.41) if agegroupT2_Engedal == "85+ years" & edu_group_engedal == "Secondary" & gender_T0==1

replace z_score_MoCA_T2 =((MoCA_T2 - 22.9)/1.96) if agegroupT2_Engedal == "85+ years" & edu_group_engedal == "Secondary" & gender_T0==2 //all women and men of all age groups for SECONDARY education

replace z_score_MoCA_T2 = ((MoCA_T2 - 26.9)/1.93) if agegroupT2_Engedal == "<75 years" & edu_group_engedal == "Tertiary" & gender_T0==1

replace z_score_MoCA_T2 =((MoCA_T2 - 26.5)/1.73) if agegroupT2_Engedal == "<75 years" & edu_group_engedal == "Tertiary" & gender_T0==2

replace z_score_MoCA_T2 = ((MoCA_T2 - 26.7)/2.00) if agegroupT2_Engedal == "75-79 years" & edu_group_engedal == "Tertiary" & gender_T0==1

replace z_score_MoCA_T2 =((MoCA_T2 - 26.5)/1.77) if agegroupT2_Engedal == "75-79 years" & edu_group_engedal == "Tertiary" & gender_T0==2

replace z_score_MoCA_T2 = ((MoCA_T2 - 26.1)/2.16) if agegroupT2_Engedal == "80-84 years" & edu_group_engedal == "Tertiary" & gender_T0==1

replace z_score_MoCA_T2 =((MoCA_T2 - 25.3)/2.12) if agegroupT2_Engedal == "80-84 years" & edu_group_engedal == "Tertiary" & gender_T0==2

replace z_score_MoCA_T2 = ((MoCA_T2 - 24.7)/2.34) if agegroupT2_Engedal == "85+ years" & edu_group_engedal == "Tertiary" & gender_T0==1

replace z_score_MoCA_T2 =((MoCA_T2 - 24.8)/1.97) if agegroupT2_Engedal == "85+ years" & edu_group_engedal == "Tertiary" & gender_T0==2 //all women and men of all age groups for TERTIARY

*adding the z-scores from previous dataset based on Borland for everyone outside the age range 70 to 90

replace z_score_MoCA_T2 = z_score_moca_st_T2 if Age_T2 <70 & z_score_MoCA_T2 !=.

sum z_score_MoCA_T2, detail

*********T3**********Generate z-scores MOCA according to Engdedal based on sex, age, education T3

gen z_score_MoCA_T3 = ((MoCA_T3 - 25.1)/2.11) if agegroupT3_Engedal == "<75 years" & edu_group_engedal == "Compulsary" & gender_T0==1 //gender 1 is female, 2 is male

replace z_score_MoCA_T3 =((MoCA_T3 - 24.3)/1.76) if agegroupT3_Engedal == "<75 years" & edu_group_engedal == "Compulsary" & gender_T0==2

```
replace z_score_MoCA_T3 = ((MoCA_T3 - 24.8)/2.22) if agegroupT3_Engedal == "75-79 years" & edu_group_engedal == "Compulsary" & gender_T0==1

replace z_score_MoCA_T3 =((MoCA_T3 - 24.4)/2.23) if agegroupT3_Engedal == "75-79 years" & edu_group_engedal == "Compulsary" & gender_T0==2

replace z_score_MoCA_T3 = ((MoCA_T3 - 24.2)/2.65) if agegroupT3_Engedal == "80-84 years" & edu_group_engedal == "Compulsary" & gender_T0==1

replace z_score_MoCA_T3 =((MoCA_T3 - 23.7)/2.28) if agegroupT3_Engedal == "80-84 years" & edu_group_engedal == "Compulsary" & gender_T0==2

replace z_score_MoCA_T3 = ((MoCA_T3 - 23.3)/2.07) if agegroupT3_Engedal == "85+ years" & edu_group_engedal == "Compulsary" & gender_T0==1

replace z_score_MoCA_T3 =((MoCA_T3 - 22.9)/2.14) if agegroupT3_Engedal == "85+ years" & edu_group_engedal == "Compulsary" & gender_T0==2   //all women and men of all age groups for COMPULSARY education


replace z_score_MoCA_T3 = ((MoCA_T3 - 25.9)/2.05) if agegroupT3_Engedal == "<75 years" & edu_group_engedal == "Secondary" & gender_T0==1

replace z_score_MoCA_T3 = ((MoCA_T3 - 25.3)/2.08) if agegroupT3_Engedal == "<75 years" & edu_group_engedal == "Secondary" & gender_T0==2

replace z_score_MoCA_T3 = ((MoCA_T3 - 25.4)/2.28) if agegroupT3_Engedal == "75-79 years" & edu_group_engedal == "Secondary" & gender_T0==1

replace z_score_MoCA_T3 =((MoCA_T3 - 25.0)/2.18) if agegroupT3_Engedal == "75-79 years" & edu_group_engedal == "Secondary" & gender_T0==2

replace z_score_MoCA_T3 = ((MoCA_T3 - 25.0)/2.26) if agegroupT3_Engedal == "80-84 years" & edu_group_engedal == "Secondary" & gender_T0==1

replace z_score_MoCA_T3 =((MoCA_T3 - 24.3)/2.13) if agegroupT3_Engedal == "80-84 years" & edu_group_engedal == "Secondary" & gender_T0==2

replace z_score_MoCA_T3 = ((MoCA_T3 - 23.7)/2.41) if agegroupT3_Engedal == "85+ years" & edu_group_engedal == "Secondary" & gender_T0==1

replace z_score_MoCA_T3 =((MoCA_T3 - 22.9)/1.96) if agegroupT3_Engedal == "85+ years" & edu_group_engedal == "Secondary" & gender_T0==2   //all women and men of all age groups for SECONDARY education


replace z_score_MoCA_T3 = ((MoCA_T3 - 26.9)/1.93) if agegroupT3_Engedal == "<75 years" & edu_group_engedal == "Tertiary" & gender_T0==1

replace z_score_MoCA_T3 =((MoCA_T3 - 26.5)/1.73) if agegroupT3_Engedal == "<75 years" & edu_group_engedal == "Tertiary" & gender_T0==2

replace z_score_MoCA_T3 = ((MoCA_T3 - 26.7)/2.00) if agegroupT3_Engedal == "75-79 years" & edu_group_engedal == "Tertiary" & gender_T0==1

replace z_score_MoCA_T3 =((MoCA_T3 - 26.5)/1.77) if agegroupT3_Engedal == "75-79 years" & edu_group_engedal == "Tertiary" & gender_T0==2

replace z_score_MoCA_T3 = ((MoCA_T3 - 26.1)/2.16) if agegroupT3_Engedal == "80-84 years" & edu_group_engedal == "Tertiary" & gender_T0==1

replace z_score_MoCA_T3 =((MoCA_T3 - 25.3)/2.12) if agegroupT3_Engedal == "80-84 years" & edu_group_engedal == "Tertiary" & gender_T0==2

replace z_score_MoCA_T3 = ((MoCA_T3 - 24.7)/2.34) if agegroupT3_Engedal == "85+ years" & edu_group_engedal == "Tertiary" & gender_T0==1
```

replace z_score_MoCA_T3 =((MoCA_T3 - 24.8)/1.97) if agegroupT3_Engedal == "85+ years" & edu_group_engedal == "Tertiary" & gender_T0==2   //all women and men of all age groups for TERTIARY

*adding the z-scores from previous dataset based on Borland for everyone outside the age range below 70

replace z_score_MoCA_T3 = z_score_moca_st_T3 if Age_T3 <70 & z_score_MoCA_T3 !=.

sum z_score_MoCA_T3, detail

************************# TMT A and B normalization based on espenes et al #1 https://www.tandfonline.com/doi/epdf/10.1080/13854046.2020.1829068?needAccess=true - age and education adjustment,

*Calculating normative performance using regression-based norms: The normative effects of demographics on performance are first determined using theregression coefficients obtained from the multiple regression analysis (Table 3)described above using the following formula

*********(Intercept + [individual age* age coeffi-cient] + [years of education * education coefficient])

*"For example, for a 60-year-oldwoman with 13 years of education, the resulting equation on TMT-B would be:([16.921] + [60 * -0.139]) + (13 * 0.170). This formula produces an individual predicted scaled score for TMT-B.

*We then subtract the scaled score obtained by the individual (Table 2) from the demographically adjusted predicted scaled score and divide by the standard deviation of the regression model residuals (Table 3) which yields standardized Z-score (Obtained scaled model score - predicted scaled score/standard deviation of the residuals obtained from the regression Z-score)"

*Generate scaled scores based on Table 2

*TMT A T1

gen Espenes_scaled_score_A_T1 = 2 if TrailA_time_T1 >70 & TrailA_time_T1 != .

replace Espenes_scaled_score_A_T1 = 3 if TrailA_time_T1 >55 & TrailA_time_T1 <71

replace Espenes_scaled_score_A_T1 = 4 if TrailA_time_T1 >63 & TrailA_time_T1 <66

replace Espenes_scaled_score_A_T1 = 5 if TrailA_time_T1 >57 & TrailA_time_T1 <64

replace Espenes_scaled_score_A_T1 = 6 if TrailA_time_T1 >53 & TrailA_time_T1 <58

replace Espenes_scaled_score_A_T1 = 7 if TrailA_time_T1 >45 & TrailA_time_T1 <54

replace Espenes_scaled_score_A_T1 = 8 if TrailA_time_T1 >39 & TrailA_time_T1 <46

replace Espenes_scaled_score_A_T1 = 9 if TrailA_time_T1 >36 & TrailA_time_T1 <40

replace Espenes_scaled_score_A_T1 = 10 if TrailA_time_T1 >32 & TrailA_time_T1 <37

replace Espenes_scaled_score_A_T1 = 11 if TrailA_time_T1 >28 & TrailA_time_T1 <33

replace Espenes_scaled_score_A_T1 = 12 if TrailA_time_T1 >26 & TrailA_time_T1 <29

replace Espenes_scaled_score_A_T1 = 13 if TrailA_time_T1 >24 & TrailA_time_T1 <27

replace Espenes_scaled_score_A_T1 = 14 if TrailA_time_T1 >21 & TrailA_time_T1 <25

replace Espenes_scaled_score_A_T1 = 15 if TrailA_time_T1 == 21

replace Espenes_scaled_score_A_T1 = 16 if TrailA_time_T1 >18 & TrailA_time_T1 <21

replace Espenes_scaled_score_A_T1 = 17 if TrailA_time_T1 >16 & TrailA_time_T1 <19

```
replace Espenes_scaled_score_A_T1 = 18 if TrailA_time_T1 ==16
replace Espenes_scaled_score_A_T1 = 19 if TrailA_time_T1 <16

*TMT A T2
gen Espenes_scaled_score_A_T2 = 2 if TrailA_time_T2 >70 & TrailA_time_T2 != .
replace Espenes_scaled_score_A_T2 = 3 if TrailA_time_T2 >55 & TrailA_time_T2 <72
replace Espenes_scaled_score_A_T2 = 4 if TrailA_time_T2 >63 & TrailA_time_T2 <66
replace Espenes_scaled_score_A_T2 = 5 if TrailA_time_T2 >57 & TrailA_time_T2 <64
replace Espenes_scaled_score_A_T2 = 6 if TrailA_time_T2 >53 & TrailA_time_T2 <58
replace Espenes_scaled_score_A_T2 = 7 if TrailA_time_T2 >45 & TrailA_time_T2 <54
replace Espenes_scaled_score_A_T2 = 8 if TrailA_time_T2 >39 & TrailA_time_T2 <46
replace Espenes_scaled_score_A_T2 = 9 if TrailA_time_T2 >36 & TrailA_time_T2 <40
replace Espenes_scaled_score_A_T2 = 10 if TrailA_time_T2 >32 & TrailA_time_T2 <37
replace Espenes_scaled_score_A_T2 = 11 if TrailA_time_T2 >28 & TrailA_time_T2 <33
replace Espenes_scaled_score_A_T2 = 12 if TrailA_time_T2 >26 & TrailA_time_T2 <29
replace Espenes_scaled_score_A_T2 = 13 if TrailA_time_T2 >24 & TrailA_time_T2 <27
replace Espenes_scaled_score_A_T2 = 14 if TrailA_time_T2 >21 & TrailA_time_T2 <25
replace Espenes_scaled_score_A_T2 = 15 if TrailA_time_T2 == 21
replace Espenes_scaled_score_A_T2 = 16 if TrailA_time_T2 >18 & TrailA_time_T2 <21
replace Espenes_scaled_score_A_T2 = 17 if TrailA_time_T2 >16 & TrailA_time_T2 <19
replace Espenes_scaled_score_A_T2 = 18 if TrailA_time_T2 ==16
replace Espenes_scaled_score_A_T2 = 19 if TrailA_time_T2 <16

*TMT A T3
gen Espenes_scaled_score_A_T3 = 2 if TrailA_time_T3 >70 & TrailA_time_T3 != .
replace Espenes_scaled_score_A_T3 = 3 if TrailA_time_T3 >55 & TrailA_time_T3 <72
replace Espenes_scaled_score_A_T3 = 4 if TrailA_time_T3 >63 & TrailA_time_T3 <66
replace Espenes_scaled_score_A_T3 = 5 if TrailA_time_T3 >57 & TrailA_time_T3 <64
replace Espenes_scaled_score_A_T3 = 6 if TrailA_time_T3 >53 & TrailA_time_T3 <58
replace Espenes_scaled_score_A_T3 = 7 if TrailA_time_T3 >45 & TrailA_time_T3 <54
replace Espenes_scaled_score_A_T3 = 8 if TrailA_time_T3 >39 & TrailA_time_T3 <46
replace Espenes_scaled_score_A_T3 = 9 if TrailA_time_T3 >36 & TrailA_time_T3 <40
replace Espenes_scaled_score_A_T3 = 10 if TrailA_time_T3 >32 & TrailA_time_T3 <37
replace Espenes_scaled_score_A_T3 = 11 if TrailA_time_T3 >28 & TrailA_time_T3 <33
replace Espenes_scaled_score_A_T3 = 12 if TrailA_time_T3 >26 & TrailA_time_T3 <29
```

replace Espenes_scaled_score_A_T3 = 13 if TrailA_time_T3 >24 & TrailA_time_T3 <27

replace Espenes_scaled_score_A_T3 = 14 if TrailA_time_T3 >21 & TrailA_time_T3 <25

replace Espenes_scaled_score_A_T3 = 15 if TrailA_time_T3 == 21

replace Espenes_scaled_score_A_T3 = 16 if TrailA_time_T3 >18 & TrailA_time_T3 <21

replace Espenes_scaled_score_A_T3 = 17 if TrailA_time_T3 >16 & TrailA_time_T3 <19

replace Espenes_scaled_score_A_T3 = 18 if TrailA_time_T3 ==16

replace Espenes_scaled_score_A_T3 = 19 if TrailA_time_T3 <16

*TMT B T1

gen Espenes_scaled_score_B_T1 = 2 if TrailB_time_T1 >165 & TrailB_time_T1 != .

replace Espenes_scaled_score_B_T1 = 3 if TrailB_time_T1 >159 & TrailB_time_T1 <166

replace Espenes_scaled_score_B_T1 = 4 if TrailB_time_T1 >154 & TrailB_time_T1 <160

replace Espenes_scaled_score_B_T1 = 5 if TrailB_time_T1 >134 & TrailB_time_T1 <155

replace Espenes_scaled_score_B_T1 = 6 if TrailB_time_T1 >120 & TrailB_time_T1 <135

replace Espenes_scaled_score_B_T1 = 7 if TrailB_time_T1 >107 & TrailB_time_T1 <121

replace Espenes_scaled_score_B_T1 = 8 if TrailB_time_T1 >95 & TrailB_time_T1 <108

replace Espenes_scaled_score_B_T1 = 9 if TrailB_time_T1 >85 & TrailB_time_T1 <96

replace Espenes_scaled_score_B_T1 = 10 if TrailB_time_T1 >77 & TrailB_time_T1 <86

replace Espenes_scaled_score_B_T1 = 11 if TrailB_time_T1 >70 & TrailB_time_T1 <78

replace Espenes_scaled_score_B_T1 = 12 if TrailB_time_T1 >62 & TrailB_time_T1 <71

replace Espenes_scaled_score_B_T1 = 13 if TrailB_time_T1 >57 & TrailB_time_T1 <63

replace Espenes_scaled_score_B_T1 = 14 if TrailB_time_T1 >50 & TrailB_time_T1 <58

replace Espenes_scaled_score_B_T1 = 15 if TrailB_time_T1 >46 & TrailB_time_T1 <51

replace Espenes_scaled_score_B_T1 = 16 if TrailB_time_T1 >41 & TrailB_time_T1 <47

replace Espenes_scaled_score_B_T1 = 17 if TrailB_time_T1 >39 & TrailB_time_T1 <42

replace Espenes_scaled_score_B_T1 = 18 if TrailB_time_T1 >33 & TrailB_time_T1 <40

replace Espenes_scaled_score_B_T1 = 19 if TrailB_time_T1 <34

*TMT B T2

gen Espenes_scaled_score_B_T2 = 2 if TrailB_time_T2 >165 & TrailB_time_T2 != .

replace Espenes_scaled_score_B_T2 = 3 if TrailB_time_T2 >159 & TrailB_time_T2 <166

replace Espenes_scaled_score_B_T2 = 4 if TrailB_time_T2 >154 & TrailB_time_T2 <160

replace Espenes_scaled_score_B_T2 = 5 if TrailB_time_T2 >134 & TrailB_time_T2 <155

```
replace Espenes_scaled_score_B_T2 = 6 if TrailB_time_T2 >120 & TrailB_time_T2 <135
replace Espenes_scaled_score_B_T2 = 7 if TrailB_time_T2 >107 & TrailB_time_T2 <121
replace Espenes_scaled_score_B_T2 = 8 if TrailB_time_T2 >95 & TrailB_time_T2 <108
replace Espenes_scaled_score_B_T2 = 9 if TrailB_time_T2 >85 & TrailB_time_T2 <96
replace Espenes_scaled_score_B_T2 = 10 if TrailB_time_T2 >77 & TrailB_time_T2 <86
replace Espenes_scaled_score_B_T2 = 11 if TrailB_time_T2 >70 & TrailB_time_T2 <78
replace Espenes_scaled_score_B_T2 = 12 if TrailB_time_T2 >62 & TrailB_time_T2 <71
replace Espenes_scaled_score_B_T2 = 13 if TrailB_time_T2 >57 & TrailB_time_T2 <63
replace Espenes_scaled_score_B_T2 = 14 if TrailB_time_T2 >50 & TrailB_time_T2 <58
replace Espenes_scaled_score_B_T2 = 15 if TrailB_time_T2 >46 & TrailB_time_T2 <51
replace Espenes_scaled_score_B_T2 = 16 if TrailB_time_T2 >41 & TrailB_time_T2 <47
replace Espenes_scaled_score_B_T2 = 17 if TrailB_time_T2 >39 & TrailB_time_T2 <42
replace Espenes_scaled_score_B_T2 = 18 if TrailB_time_T2 >33 & TrailB_time_T2 <40
replace Espenes_scaled_score_B_T2 = 19 if TrailB_time_T2 <34
```

```
*TMT B T3
gen Espenes_scaled_score_B_T3 = 2 if TrailB_time_T3 >165 & TrailB_time_T3 != .
replace Espenes_scaled_score_B_T3 = 3 if TrailB_time_T3 >159 & TrailB_time_T3 <166
replace Espenes_scaled_score_B_T3 = 4 if TrailB_time_T3 >154 & TrailB_time_T3 <160
replace Espenes_scaled_score_B_T3 = 5 if TrailB_time_T3 >134 & TrailB_time_T3 <155
replace Espenes_scaled_score_B_T3 = 6 if TrailB_time_T3 >120 & TrailB_time_T3 <135
replace Espenes_scaled_score_B_T3 = 7 if TrailB_time_T3 >107 & TrailB_time_T3 <121
replace Espenes_scaled_score_B_T3 = 8 if TrailB_time_T3 >95 & TrailB_time_T3 <108
replace Espenes_scaled_score_B_T3 = 9 if TrailB_time_T3 >85 & TrailB_time_T3 <96
replace Espenes_scaled_score_B_T3 = 10 if TrailB_time_T3 >77 & TrailB_time_T3 <86
replace Espenes_scaled_score_B_T3 = 11 if TrailB_time_T3 >70 & TrailB_time_T3 <78
replace Espenes_scaled_score_B_T3 = 12 if TrailB_time_T3 >62 & TrailB_time_T3 <71
replace Espenes_scaled_score_B_T3 = 13 if TrailB_time_T3 >57 & TrailB_time_T3 <63
replace Espenes_scaled_score_B_T3 = 14 if TrailB_time_T3 >50 & TrailB_time_T3 <58
replace Espenes_scaled_score_B_T3 = 15 if TrailB_time_T3 >46 & TrailB_time_T3 <51
replace Espenes_scaled_score_B_T3 = 16 if TrailB_time_T3 >41 & TrailB_time_T3 <47
replace Espenes_scaled_score_B_T3 = 17 if TrailB_time_T3 >39 & TrailB_time_T3 <42
replace Espenes_scaled_score_B_T3 = 18 if TrailB_time_T3 >33 & TrailB_time_T3 <40
replace Espenes_scaled_score_B_T3 = 19 if TrailB_time_T3 <34
```

*TMT A equation conversion z-scores

gen z_score_TMT_A_Espenes_T1 = (Espenes_scaled_score_A_T1 - (19.437 + Age_T1 * -0.144)) /2.675 // T1 TMT A

gen z_score_TMT_A_Espenes_T2 = (Espenes_scaled_score_A_T2 - (19.437 + Age_T2 * -0.144)) /2.675 // T2 TMT A

gen z_score_TMT_A_Espenes_T3 = (Espenes_scaled_score_A_T3 - (19.437 + Age_T3 * -0.144)) /2.675 // T3 TMT A

*TMT B equation conversion z-scores

gen z_score_TMT_B_Espenes_T1 = (Espenes_scaled_score_B_T1 - ((16.921 + Age_T1 * -0.139)+ (education_year_T0*0.170))) / 2.645

gen z_score_TMT_B_Espenes_T2 = (Espenes_scaled_score_B_T2 - ((16.921 + Age_T2 * -0.139)+ (education_year_T0*0.170))) / 2.645

gen z_score_TMT_B_Espenes_T3 = (Espenes_scaled_score_B_T3 - ((16.921 + Age_T3 * -0.139)+ (education_year_T0*0.170))) / 2.645

*********************************# 10 word test CERAD normalization #2

* edu_group_engedal works for the definition of education groups in CERAD Wagle as well: education (categorized as compulsory (≤10 years), secondary (11–13 years) and tertiary (≥14 years))

*Creating z-scores based on Wagle et al. 2023, https://www.aldringoghelse.no/normkalkulatorer/normkalkulator-for-cerad-tiordstest/

***Using the regression equation:The z-score can be calculated as the difference between the observed subtest score and the subtest score estimated from the regression models in Table 3, divided by the model's root mean square error (RMSE).

*model's root mean square error (RMSE) == 1.597

*T1

gen z_score_recall10_delayed_T1 = ((recall10_delayed_T1 - (13.46 + (Age_T1* -0.1) + 0.7))/1.597) if edu_group_engedal == "Tertiary" & gender_T0==1   // for tertiary education and woman

replace z_score_recall10_delayed_T1 = ((recall10_delayed_T1 - (13.46 + (Age_T1* -0.1)))/1.597) if edu_group_engedal == "Secondary" & gender_T0==1 // for secondary education and woman

replace z_score_recall10_delayed_T1 = ((recall10_delayed_T1 - (13.46 + (Age_T1* -0.1) -0.14))/1.597) if edu_group_engedal == "Compulsary" & gender_T0==1 // for primary/compulsary education and woman

replace z_score_recall10_delayed_T1 = ((recall10_delayed_T1 - (13.46 + (Age_T1* -0.1) + 0.7 -3.11))/1.597) if edu_group_engedal == "Tertiary" & gender_T0==2   // for tertiary education and man

replace z_score_recall10_delayed_T1 = ((recall10_delayed_T1 - (13.46 + (Age_T1* -0.1) -3.11))/1.597) if edu_group_engedal == "Secondary" & gender_T0==2 // for secondary education and man

replace z_score_recall10_delayed_T1 = ((recall10_delayed_T1 - (13.46 + (Age_T1* -0.1) -0.14 -3.11))/1.597) if edu_group_engedal == "Compulsary" & gender_T0==2 // for primary/compulsary education and man

*T2

gen z_score_recall10_delayed_T2 = ((recall10_delayed_T2 - (13.46 + (Age_T2* -0.1) + 0.7))/1.597) if edu_group_engedal == "Tertiary" & gender_T0==1   // for tertiary education and woman

replace z_score_recall10_delayed_T2 = ((recall10_delayed_T2 - (13.46 + (Age_T2* -0.1)))/1.597) if edu_group_engedal == "Secondary" & gender_T0==1 // for secondary education and woman

replace z_score_recall10_delayed_T2 = ((recall10_delayed_T2 - (13.46 + (Age_T2* -0.1) -0.14))/1.597) if edu_group_engedal == "Compulsary" & gender_T0==1 // for primary/compulsary education and woman

replace z_score_recall10_delayed_T2 = ((recall10_delayed_T2 - (13.46 + (Age_T2* -0.1) + 0.7 -3.11))/1.597) if edu_group_engedal == "Tertiary" & gender_T0==2 // for tertiary education and man

replace z_score_recall10_delayed_T2 = ((recall10_delayed_T2 - (13.46 + (Age_T2* -0.1) -3.11))/1.597) if edu_group_engedal == "Secondary" & gender_T0==2 // for secondary education and man

replace z_score_recall10_delayed_T2 = ((recall10_delayed_T2 - (13.46 + (Age_T2* -0.1) -0.14 -3.11))/1.597) if edu_group_engedal == "Compulsary" & gender_T0==2 // for primary/compulsary education and man

*T3

gen z_score_recall10_delayed_T3 = ((recall10_delayed_T3 - (13.46 + (Age_T3* -0.1) + 0.7))/1.597) if edu_group_engedal == "Tertiary" & gender_T0==1 // for tertiary education and woman

replace z_score_recall10_delayed_T3 = ((recall10_delayed_T3 - (13.46 + (Age_T3* -0.1)))/1.597) if edu_group_engedal == "Secondary" & gender_T0==1 // for secondary education and woman

replace z_score_recall10_delayed_T3 = ((recall10_delayed_T3 - (13.46 + (Age_T3* -0.1) -0.14))/1.597) if edu_group_engedal == "Compulsary" & gender_T0==1 // for primary/compulsary education and woman

replace z_score_recall10_delayed_T3 = ((recall10_delayed_T3 - (13.46 + (Age_T3* -0.1) + 0.7 -3.11))/1.597) if edu_group_engedal == "Tertiary" & gender_T0==2 // for tertiary education and man

replace z_score_recall10_delayed_T3 = ((recall10_delayed_T3 - (13.46 + (Age_T3* -0.1) -3.11))/1.597) if edu_group_engedal == "Secondary" & gender_T0==2 // for secondary education and man

replace z_score_recall10_delayed_T3 = ((recall10_delayed_T3 - (13.46 + (Age_T3* -0.1) -0.14 -3.11))/1.597) if edu_group_engedal == "Compulsary" & gender_T0==2 // for primary/compulsary education and man

sum z_score_recall10_delayed_T1

sum z_score_recall10_delayed_T2

sum z_score_recall10_delayed_T3

replace z_score_recall10_delayed_T1 = z_score_delayed_stina_T1 if Age_T1 <70 & Age_T1 >59

replace z_score_recall10_delayed_T2 = z_score_delayed_stina_T2 if Age_T2 <70 & Age_T2 >59

replace z_score_recall10_delayed_T3 = z_score_delayed_stina_T3 if Age_T3 <70 & Age_T3 >59

gen edu_group_luck = 1 if education_year_T0 < 15

replace edu_group_luck = 2 if education_year_T0 >14 & education_year_T0 !=.

tab edu_group_luck

replace z_score_recall10_delayed_T1 = ((recall10_delayed_T1 - 8.2)/1.5) if Age_T1 <60 & gender_T0==1 & edu_group_luck ==1 // a woman with no university degree younger 60 taken from age group 60 to 64, Table 2 Luck et al 2018

replace z_score_recall10_delayed_T1 = ((recall10_delayed_T1 - 8.3)/1.4) if Age_T1 <60 & gender_T0==1 & edu_group_luck ==2 // a woman with university degree younger 60 taken from age group 60 to 64, Table 2 Luck et al 2018

```
replace  z_score_recall10_delayed_T1 = ((recall10_delayed_T1 - 6.8)/2)   if Age_T1 <60 & gender_T0==2 &  edu_group_luck ==1  // a man with no university degree younger 60 taken from age group 60 to 64, Table 2 Luck et al 2018

replace  z_score_recall10_delayed_T1 = ((recall10_delayed_T1 - 7.5)/1.7)   if Age_T1 <60 & gender_T0==2 &  edu_group_luck ==2  // a man with university degree younger 60 taken from age group 60 to 64, Table 2 Luck et al 2018


sum z_score_MoCA_T1 if meanstep1 !=.   //MoCA when step not missing

sum z_score_MoCA_T2 if meanstep2 !=.

sum z_score_MoCA_T3 if meanstep3 !=.

sum z_score_TMT_A_Espenes_T1 if meanstep1 !=.

sum z_score_TMT_A_Espenes_T2 if meanstep2 !=.

sum z_score_TMT_A_Espenes_T3 if meanstep3 !=.

sum z_score_TMT_B_Espenes_T1 if meanstep1 !=.

sum z_score_TMT_B_Espenes_T2 if meanstep2 !=.

sum z_score_TMT_B_Espenes_T3 if meanstep3 !=.

sum z_score_recall10_delayed_T1 if meanstep1 !=.

sum z_score_recall10_delayed_T2 if meanstep2 !=.

sum z_score_recall10_delayed_T3 if meanstep3 !=.


sum z_score_MoCA_T1 if meanstep1 !=. & z_score_TMT_A_Espenes_T1 !=. & z_score_TMT_B_Espenes_T1 !=. & z_score_recall10_delayed_T1 !=.

sum z_score_MoCA_T2 if meanstep2 !=. & z_score_TMT_A_Espenes_T2 !=. & z_score_TMT_B_Espenes_T2 !=. & z_score_recall10_delayed_T2 !=.

sum z_score_MoCA_T3 if meanstep3 !=. & z_score_TMT_A_Espenes_T3 !=. & z_score_TMT_B_Espenes_T3 !=. & z_score_recall10_delayed_T3 !=.


**Look into missingness more:

preserve

keep if include==1

misstable summarize meanstep1 meanstep2 meanstep3 z_score_MoCA_T1 z_score_MoCA_T2 z_score_MoCA_T3 z_score_TMT_A_Espenes_T1 z_score_TMT_A_Espenes_T2 z_score_TMT_A_Espenes_T3 z_score_TMT_B_Espenes_T1 z_score_TMT_B_Espenes_T2 z_score_TMT_B_Espenes_T3 z_score_recall10_delayed_T1 z_score_recall10_delayed_T2 z_score_recall10_delayed_T3

restore


label define TOAST_classification_T0 1 "Arteriosclerosis" 2 "Cardiac emboli" 3 "Small vessel disease" 4 "Other etiology" 5 "Undetermined", replace

stddiff Age_T0 MRS_T1 NIHSS1 education_y i.gender_T0 i.living_alone_T0 HADSd_T1 i.lesion_type_T0 i.TOAST_classification_T0 i.diabetes_prestroke_T0 bmi_baseline, by(include)


***** IPW *****
```

```
logistic include i.agegroupT0 i.edu_group_eng i.mrsgroup2   // highest ipw 4.3


preserve
        // Logistisk regresjonsmodell hvor sample er brukt for deltakelse
predict yhat
                                                                        // Prediction for outcome
gen ipw=1/yhat
                                                        // Generate IPW
label variable ipw "IPW"
replace ipw =1 if include==0
sum ipw
hist ipw if include ==1
*keep if include ==1   // only to  control
*collapse (sum) ipw   / the number of people you're trying to represent with your weighted sample.
*list ipw


hist ipw if include==1
sum ipw if include==1, detail


*trimming the IPW to 97.5 and 2.5th percentiles
preserve
keep if include==1
_pctile ipw, p(3 97)
local p3 = r(r1)
local p97 = r(r2)


// Optional: Check what they are
di "3rd percentile: `p3'"
di "97th percentile: `p97'"
restore


gen ipw_trimmed = ipw if include==1
replace ipw_trimmed = r(r2) if ipw > r(r2) & include==1
hist ipw_trimmed if include==1
sum ipw_trimmed, detail
*compare
histogram ipw if include==1, width(0.1) start(1) frequency
```

```
histogram ipw_trimmed if include==1, width(0.4) start(1) frequency


collapse (min) ipw_trimmed, by( mrsgroup2 agegroupT0 edu_group_eng)

sort agegroupT0 mrsgroup

cd "C:\Users\.....

save ipw_weights_art3, replace

restore
```

Code in R studio

The code used to create the RI-CLPM was generated by Jeroen D. Mulder and Ellen Hamaker [9] **(see: https://jeroendmulder.github.io/RI-CLPM/lavaan.html)** and is based on the lavaan package in Rstudio.[10]

```
#if you need to install anything, uncomment the below install lines for now

#install.packages('lavaan')

#install.packages('tidyverse')

require(lavaan)

require(tidyverse)


# For all calculations use:

library(haven)

Main_varaibles_backup_for_R <- read_dta("M:/…")

View(Main_varaibles_backup_for_R)


##### The relation of MoCA and step count over time in Nor-COAST

# Some descriptives, Note: For software to easier reach convergence Steps are scaled by 1000: 1. This brings expsore and bnormalized outcome variables closer.

Main_varaibles…%>%

  select(x1, x2, x3,y1,y2, y3) %>%

  mutate(pid = 1:n()) %>%

  gather(key, value, -pid) %>%

  extract(col = key, into = c('var', 'wave'), regex = '(\\w)(\\d)') %>%

  ggplot(aes(x = value)) +

  geom_density(alpha = 1) +

  facet_grid(wave~var, scales = 'free') +
```

```
  theme_classic()


Main_varaibles_...
  select(x1, x2, x3,y1,y2, y3) %>%
  mutate(pid = 1:n()) %>%
  gather(key, value, -pid) %>%
  extract(col = key, into = c('var', 'wave'), regex = '(\\w)(\\d)')

Main_varaibles_...
  ggplot(aes(x = wave, y = value, color = var, group = var)) +
  geom_point(position = position_jitter(w = .2), alpha = .1) +
  geom_line(stat = 'identity', aes(group = interaction(var, pid)), alpha = .04) +
  geom_line(stat = 'smooth', method = 'lm', size = 1) +
  theme_classic()

riclpmModel <-
  '
#Note, the data contain x1-3 and y1-3
#Latent mean Structure with intercepts

kappa =~ 1*x1 + 1*x2 + 1*x3
omega =~ 1*y1 + 1*y2 + 1*y3

x1 ~ mu1*1 #intercepts
x2 ~ mu2*1
x3 ~ mu3*1
y1 ~ pi1*1
y2 ~ pi2*1
y3 ~ pi3*1

kappa ~~ kappa #variance
omega ~~ omega #variance
kappa ~~ omega #covariance
```

```
#laten vars for AR and cross-lagged effects
p1 =~ 1*x1 #each factor loading set to 1
p2 =~ 1*x2
p3 =~ 1*x3
q1 =~ 1*y1
q2 =~ 1*y2
q3 =~ 1*y3
p3 ~ alpha3*p2 + beta3*q2
p2 ~ alpha2*p1 + beta2*q1

q3 ~ delta3*q2 + gamma3*p2
q2 ~ delta2*q1 + gamma2*p1

p1 ~~ p1 #variance
p2 ~~ u2*p2
p3 ~~ u3*p3
q1 ~~ q1 #variance
q2 ~~ v2*q2
q3 ~~ v3*q3

p1 ~~ q1 #p1 and q1 covariance
p2 ~~ q2 #p2 and q2 covariance
p3 ~~ q3 #p2 and q2 covariance'

fit <- lavaan(riclpmModel, data = Main_varaibles_...
        missing = 'ML', #for the missing data!
        int.ov.free = F,
        int.lv.free = F,
        auto.fix.first = F,
        auto.fix.single = F,
        auto.cov.lv.x = F,
        auto.cov.y = F,
        auto.var = F)

summary(fit, standardized = T)
```

```
standardizedsolution(fit)

#How are the random intercepts distributed?

fscores <- lavPredict(fit)
library(ggplot2)

fscores_df <- as.data.frame(fscores)
fscores_df$pid <- 1:nrow(fscores_df)

# Example: plot distribution of RIx
ggplot(fscores_df, aes(x = kappa)) +
  geom_histogram(bins = 30, fill = "skyblue", color = "black") +
  theme_minimal() +
  labs(title = "Distribution of Random Intercept for X",
       x = "Random Intercept (RIx)", y = "Frequency")

# Example: scatterplot of RIx vs RIy
ggplot(fscores_df, aes(x = kappa, y = omega)) +
  geom_point(alpha = 0.6) +
  theme_minimal() +
  labs(title = "Random Intercepts: X vs Y", x = "RIx", y = "RIy")

# add colours to the scatter depending on how many observation go into the model per participant
library(dplyr)

# Count number of timepoints per pid (any value for x or y at that wave)
obs_count <- Main_varaibles_...%>%
  group_by(pid, wave) %>%
  summarise(has_data = any(!is.na(value)), .groups = "drop") %>%
  group_by(pid) %>%
  summarise(n_timepoints = sum(has_data)) %>%
  mutate(contribution = case_when(
    n_timepoints == 3 ~ "All 3 time points",
    n_timepoints == 2 ~ "2 time points",
```

```
    n_timepoints == 1 ~ "1 time point"
  ))

fscores <- lavPredict(fit)
fscores_df <- as.data.frame(fscores)
fscores_df$pid <- 1:nrow(fscores_df)

# Join the contribution info
fscores_df <- fscores_df %>%
  left_join(obs_count, by = "pid")



#Scatter
ggplot(fscores_df, aes(x = kappa, y = omega, color = contribution)) +
  geom_point(alpha = 0.7) +
  theme_minimal() +
  labs(title = "Random Intercepts Colored by Data Contribution",
       x = "RIx", y = "RIy", color = "Data Contribution")
# # Scatter 2.0 just bigger
ggplot(fscores_df, aes(x = kappa, y = omega, color = contribution)) +
  geom_point(alpha = 0.7) +
  theme_minimal(base_size = 18) +  # increases overall text size
  labs(
    title = "Random Intercepts of step count (x) and MoCA z-score (y)",
    x = "RIx",
    y = "RIy",
    color = "Data Contribution"
  ) +
  theme(
    plot.title = element_text(size = 20, face = "bold", hjust = 0.5),
    axis.title = element_text(size = 20),
    axis.text = element_text(size = 20),
    legend.title = element_text(size = 20),
    legend.text = element_text(size = 20)
  )
```

```
riclpmModeladjusted <-
  '
#Note, the data contain x1-3 and y1-3
#Latent mean Structure with intercepts

kappa =~ 1*x1 + 1*x2 + 1*x3
omega =~ 1*y1 + 1*y2 + 1*y3

#remove measurement error as recommednded by hamaker for smaller samples and not many waves? doesnt change results at all
#x1 ~~ 0*x1
#x2 ~~ 0*x2
#x3 ~~ 0*x3
#y1 ~~ 0*y1
#y2 ~~ 0*y2
#y3 ~~ 0*y3

x1 ~ mu1*1 #intercepts
x2 ~ mu2*1
x3 ~ mu3*1
y1 ~ pi1*1
y2 ~ pi2*1
y3 ~ pi3*1

kappa ~~ kappa #variance
omega ~~ omega #variance
kappa ~~ omega #covariance

#laten vars for AR and cross-lagged effects
p1 =~ 1*x1 #each factor loading set to 1
p2 =~ 1*x2
p3 =~ 1*x3
q1 =~ 1*y1
q2 =~ 1*y2
q3 =~ 1*y3
```

```
# Regression of random intercepts on covariates
kappa + omega ~ stroke + sex + edu + age +liv

# Add as a sensitivity only : Regression on observed variables
 # Regression of observed variables on z1 (constrained)
  #x1 + x2 + x3  ~ s1* sex + age+ stroke + liv + stroke  # Constrained over time.
  #y1 + y2 + y3  ~ s2* sex + age+ stroke + liv +stroke # Constrained over time.

p3 ~ alpha3*p2 + beta3*q2
p2 ~ alpha2*p1 + beta2*q1

q3 ~ delta3*q2 + gamma3*p2
q2 ~ delta2*q1 + gamma2*p1

p1 ~~ p1 #variance
p2 ~~ u2*p2
p3 ~~ u3*p3
q1 ~~ q1 #variance
q2 ~~ v2*q2
q3 ~~ v3*q3

p1 ~~ q1 #p1 and q1 covariance
p2 ~~ q2 #p2 and q2 covariance
p3 ~~ q3 #p2 and q2 covariance'

fitadjusted <- lavaan(riclpmModeladjusted, data = Main_varaibles_...
        missing = 'ML', #for the missing data!
        sampling.weights = "ipw_trimmed",     ##   OBS!!! added to check, run with and without excel file under phd/artcile 
3/folder heather/ipw
        int.ov.free = F,
        int.lv.free = F,
        auto.fix.first = F,
        auto.fix.single = F,
        auto.cov.lv.x = F,
        auto.cov.y = F,
```

```
        auto.var = F)

summary(fitadjusted, standardized = T)

standardizedSolution(fitadjusted)


#       TMT A


# Some descriptives, Note: For software to easier reach convergence Steps are scaled by 1000: 1. This brings expsore and bnormalized outcome variables closer.



riclpmModelTMTA <-
 '

#Note, the data contain x1-3 and a1-3

#Latent mean Structure with intercepts


kappa =~ 1*x1 + 1*x2 + 1*x3

omega =~ 1*a1 + 1*a2 + 1*a3


x1 ~ mu1*1 #intercepts

x2 ~ mu2*1

x3 ~ mu3*1

a1 ~ pi1*1

a2 ~ pi2*1

a3 ~ pi3*1


kappa ~~ kappa #variance

omega ~~ omega #variance

kappa ~~ omega #covariance



#laten vars for AR and cross-lagged effects

p1 =~ 1*x1 #each factor loading set to 1

p2 =~ 1*x2

p3 =~ 1*x3

q1 =~ 1*a1

q2 =~ 1*a2
```

```
q3 =~ 1*a3

p3 ~ alpha3*p2 + beta3*q2
p2 ~ alpha2*p1 + beta2*q1

q3 ~ delta3*q2 + gamma3*p2
q2 ~ delta2*q1 + gamma2*p1

p1 ~~ p1 #variance
p2 ~~ u2*p2
p3 ~~ u3*p3
q1 ~~ q1 #variance
q2 ~~ v2*q2
q3 ~~ v3*q3

p1 ~~ q1 #p1 and q1 covariance
p2 ~~ q2 #p2 and q2 covariance
p3 ~~ q3 #p2 and q2 covariance'

fitTMTA <- lavaan(riclpmModelTMTA, data = Main_varaibles_...
        missing = 'ML', #for the missing data!
        int.ov.free = F,
        int.lv.free = F,
        auto.fix.first = F,
        auto.fix.single = F,
        auto.cov.lv.x = F,
        auto.cov.y = F,
        auto.var = F)
summary(fitTMTA, standardized = T)
standardizedsolution(fitTMTA)

#How are the random intercepts distributed?

fscorestmta <- lavPredict(fitTMTA)
library(ggplot2)
```

```
fscorestmta_df <- as.data.frame(fscorestmta)
fscorestmta_df$pid <- 1:nrow(fscorestmta_df)

# Example: plot distribution of RIx
ggplot(fscorestmta_df, aes(x = kappa)) +
  geom_histogram(bins = 30, fill = "skyblue", color = "black") +
  theme_minimal() +
  labs(title = "Distribution of Random Intercept for X",
       x = "Random Intercept (RIx)", a = "Frequency")

# Example: scatterplot of RIx vs RIy
ggplot(fscorestmta_df, aes(x = kappa, y = omega)) +
  geom_point(alpha = 0.6) +
  theme_minimal() +
  labs(title = "Random Intercepts: X vs Y", x = "RIx", y = "RIy")

# Now I want to add colours to the scatter dependeing on how mamny observation go into the model per participant
library(dplyr)

# Count number of timepoints per pid (any value for x or y at that wave)
obs_count <- Main_varaibles_... %>%
  group_by(pid, wave) %>%
  summarise(has_data = any(!is.na(value)), .groups = "drop") %>%
  group_by(pid) %>%
  summarise(n_timepoints = sum(has_data)) %>%
  mutate(contribution = case_when(
    n_timepoints == 3 ~ "All 3 time points",
    n_timepoints == 2 ~ "2 time points",
    n_timepoints == 1 ~ "1 time point"
  ))

fscorestmta <- lavPredict(fitTMTA)
fscorestmta_df <- as.data.frame(fscorestmta)
fscorestmta_df$pid <- 1:nrow(fscorestmta_df)
```

```
# Join the contribution info
fscorestmta_df <- fscorestmta_df %>%
  left_join(obs_count, by = "pid")

#Scatter
ggplot(fscorestmta_df, aes(x = kappa, y = omega, color = contribution)) +
  geom_point(alpha = 0.7) +
  theme_minimal() +
  labs(title = "Random Intercepts Colored by Data Contribution",
       x = "RIx", y = "RIy", color = "Data Contribution")
# # Scatter 2.0 just bigger
ggplot(fscorestmta_df, aes(x = kappa, y = omega, color = contribution)) +
  geom_point(alpha = 0.7) +
  theme_minimal(base_size = 18) +  # increases overall text size
  labs(
    title = "Random Intercepts of step count (x) and TMT-A z-score (Y)",
    x = "RIx",
    y = "RIy",
    color = "Data Contribution"
  ) +
  theme(
    plot.title = element_text(size = 20, face = "bold", hjust = 0.5),
    axis.title = element_text(size = 20),
    axis.text = element_text(size = 20),
    legend.title = element_text(size = 20),
    legend.text = element_text(size = 20)
  )

# Adjsuted for age, sex, education, stroke severity, depression and living condition

riclpm_TMTA_adjusted <-
  '
#Note, the data contain x1-3 and a1-3
#Latent mean Structure with intercepts
```

```
kappa =~ 1*x1 + 1*x2 + 1*x3

omega =~ 1*a1 + 1*a2 + 1*a3

x1 ~ mu1*1 #intercepts

x2 ~ mu2*1

x3 ~ mu3*1

a1 ~ pi1*1

a2 ~ pi2*1

a3 ~ pi3*1

kappa ~~ kappa #variance

omega ~~ omega #variance

kappa ~~ omega #covariance

#laten vars for AR and cross-lagged effects

p1 =~ 1*x1 #each factor loading set to 1

p2 =~ 1*x2

p3 =~ 1*x3

q1 =~ 1*a1

q2 =~ 1*a2

q3 =~ 1*a3

# Regression of random intercepts on z1

kappa + omega ~ stroke + sex + edu + age +liv

# Add as a sensitivity: Regression on observed variables

 # Regression of observed variables on z1 (constrained)

#  x1 + x2 + x3  ~ s1* sex + age+ stroke + liv + stroke  # Constrained over time.

 # a1 + a2 + a3  ~ s2* sex + age+ stroke + liv +stroke # Constrained over time.

p3 ~ alpha3*p2 + beta3*q2

p2 ~ alpha2*p1 + beta2*q1
```

```
q3 ~ delta3*q2 + gamma3*p2
q2 ~ delta2*q1 + gamma2*p1

p1 ~~ p1 #variance
p2 ~~ u2*p2
p3 ~~ u3*p3
q1 ~~ q1 #variance
q2 ~~ v2*q2
q3 ~~ v3*q3

p1 ~~ q1 #p1 and q1 covariance
p2 ~~ q2 #p2 and q2 covariance
p3 ~~ q3 #p2 and q2 covariance'

fitTMTAadjusted <- lavaan(riclpm_TMTA_adjusted, data = Main_varaibles_...,
        missing = 'ML', #for the missing data!
        sampling.weights = "ipw_trimmed",    ## check, run with and without excel file under phd/artcile 3/folder heather/ipw
        int.ov.free = F,
        int.lv.free = F,
        auto.fix.first = F,
        auto.fix.single = F,
        auto.cov.lv.x = F,
        auto.cov.y = F,
        auto.var = F)
summary(fitTMTAadjusted, standardized = T)
standardizedsolution(fitTMTAadjusted)
fitmeasures(fitTMTAadjusted)

###########################################################################################

#    TMT_B

riclpmModelTMTb <-
  '
#Note, the data contain x1-3 and a1-3
```

```
#Latent mean Structure with intercepts

kappa =~ 1*x1 + 1*x2 + 1*x3
omega =~ 1*b1 + 1*b2 + 1*b3

x1 ~ mu1*1 #intercepts
x2 ~ mu2*1
x3 ~ mu3*1
b1 ~ pi1*1
b2 ~ pi2*1
b3 ~ pi3*1

kappa ~~ kappa #variance
omega ~~ omega #variance
kappa ~~ omega #covariance

#laten vars for AR and cross-lagged effects
p1 =~ 1*x1 #each factor loading set to 1
p2 =~ 1*x2
p3 =~ 1*x3
q1 =~ 1*b1
q2 =~ 1*b2
q3 =~ 1*b3

p3 ~ alpha3*p2 + beta3*q2
p2 ~ alpha2*p1 + beta2*q1

q3 ~ delta3*q2 + gamma3*p2
q2 ~ delta2*q1 + gamma2*p1

p1 ~~ p1 #variance
p2 ~~ u2*p2
p3 ~~ u3*p3
q1 ~~ q1 #variance
q2 ~~ v2*q2
```

```
q3 ~~ v3*q3

p1 ~~ q1 #p1 and q1 covariance
p2 ~~ q2 #p2 and q2 covariance
p3 ~~ q3 #p2 and q2 covariance'

fitTMTb <- lavaan(riclpmModelTMTb, data = Main_varaibles_...,
                  missing = 'ML', #for the missing data!
                  int.ov.free = F,
                  int.lv.free = F,
                  auto.fix.first = F,
                  auto.fix.single = F,
                  auto.cov.lv.x = F,
                  auto.cov.y = F,
                  auto.var = F)
summary(fitTMTb, standardized = T)

standardizedSolution(fitTMTb)  #this gives the same as the stata output

#How are the random intercepts distributed? For Heather

fscorestmtb <- lavPredict(fitTMTb)
library(ggplot2)

fscorestmtb_df <- as.data.frame(fscorestmtb)
fscorestmtb_df$pid <- 1:nrow(fscorestmtb_df)

# Example: plot distribution of RIx
ggplot(fscorestmtb_df, aes(x = kappa)) +
  geom_histogram(bins = 30, fill = "skyblue", color = "black") +
  theme_minimal() +
  labs(title = "Distribution of Random Intercept for X",
       x = "Random Intercept (RIx)", y = "Frequency")

# Example: scatterplot of RIx vs RIy
```

```
ggplot(fscorestmtb_df, aes(x = kappa, y = omega)) +
  geom_point(alpha = 0.6) +
  theme_minimal() +
  labs(title = "Random Intercepts: X vs Y", x = "RIx", y = "RIy")

# Now I want to add colours to the scatter dependeing on how mamny observation go into the model per participant
library(dplyr)

# Count number of timepoints per pid (any value for x or y at that wave)
obs_count <- Main_varaibles_... %>%
  group_by(pid, wave) %>%
  summarise(has_data = any(!is.na(value)), .groups = "drop") %>%
  group_by(pid) %>%
  summarise(n_timepoints = sum(has_data)) %>%
  mutate(contribution = case_when(
    n_timepoints == 3 ~ "All 3 time points",
    n_timepoints == 2 ~ "2 time points",
    n_timepoints == 1 ~ "1 time point"
  ))

fscorestmtb <- lavPredict(fitTMTb)
fscorestmtb_df <- as.data.frame(fscorestmtb)
fscorestmtb_df$pid <- 1:nrow(fscorestmtb_df)

# Join the contribution info
fscorestmtb_df <- fscorestmtb_df %>%
  left_join(obs_count, by = "pid")

#Scatter
ggplot(fscorestmtb_df, aes(x = kappa, y = omega, color = contribution)) +
  geom_point(alpha = 0.7) +
  theme_minimal() +
  labs(title = "Random Intercepts Colored by Data Contribution",
       x = "RIx", y = "RIy", color = "Data Contribution")
```

```
ggplot(fscorestmtb_df, aes(x = kappa, y = omega, color = contribution)) +
  geom_point(alpha = 0.7) +
  theme_minimal(base_size = 18) +  # increases overall text size
  labs(
    title = "Random Intercepts of step count (x) and TMT-B z-score (Y)",
    x = "RIx",
    y = "RIy",
    color = "Data Contribution"
  ) +
  theme(
    plot.title = element_text(size = 20, face = "bold", hjust = 0.5),
    axis.title = element_text(size = 20),
    axis.text = element_text(size = 20),
    legend.title = element_text(size = 20),
    legend.text = element_text(size = 20)
  )


#Now get an adjusted RI-CLPM

riclpmModelTMTbadjusted <-
  '
#Note, the data contain x1-3 and a1-3
#Latent mean Structure with intercepts

kappa =~ 1*x1 + 1*x2 + 1*x3
omega =~ 1*b1 + 1*b2 + 1*b3

x1 ~ mu1*1 #intercepts
x2 ~ mu2*1
x3 ~ mu3*1
b1 ~ pi1*1
b2 ~ pi2*1
b3 ~ pi3*1
```

```
kappa ~~ kappa #variance
omega ~~ omega #variance
kappa ~~ omega #covariance

#laten vars for AR and cross-lagged effects
p1 =~ 1*x1 #each factor loading set to 1
p2 =~ 1*x2
p3 =~ 1*x3
q1 =~ 1*b1
q2 =~ 1*b2
q3 =~ 1*b3

# Regression of random intercepts on z1
kappa + omega ~ stroke + sex + edu + age +liv

# Add as a sensitivity: Regression on observed variables
 # Regression of observed variables on z1 (constrained)
 # x1 + x2 + x3  ~ s1* sex + age+ stroke + liv + stroke  # Constrained over time.
 # b1 + b2 + b3  ~ s2* sex + age+ stroke + liv +stroke # Constrained over time.

p3 ~ alpha3*p2 + beta3*q2
p2 ~ alpha2*p1 + beta2*q1

q3 ~ delta3*q2 + gamma3*p2
q2 ~ delta2*q1 + gamma2*p1

p1 ~~ p1 #variance
p2 ~~ u2*p2
p3 ~~ u3*p3
q1 ~~ q1 #variance
q2 ~~ v2*q2
q3 ~~ v3*q3

p1 ~~ q1 #p1 and q1 covariance
p2 ~~ q2 #p2 and q2 covariance
```

```
p3 ~~ q3 #p2 and q2 covariance'

fitTMTbadjusted <- lavaan(riclpmModelTMTbadjusted, data = Main_varaibles_backup_for_R,
                    sampling.weights = "ipw_trimmed",     ## run with and without
              missing = 'ML', #for the missing data!
              int.ov.free = F,
              int.lv.free = F,
              auto.fix.first = F,
              auto.fix.single = F,
              auto.cov.lv.x = F,
              auto.cov.y = F,
              auto.var = F)
summary(fitTMTbadjusted, standardized = T)

standardizedSolution(fitTMTbadjusted)
fitmeasures(fitTMTbadjusted)

##########################################################################################################
#DELAYED RECALL
riclpmModelrecall <-
  '
#Note, the data contain x1-3 and a1-3
#Latent mean Structure with intercepts

kappa =~ 1*x1 + 1*x2 + 1*x3
omega =~ 1*d1 + 1*d2 + 1*d3

x1 ~ mu1*1 #intercepts
x2 ~ mu2*1
x3 ~ mu3*1
d1 ~ pi1*1
d2 ~ pi2*1
d3 ~ pi3*1

kappa ~~ kappa #variance
```

```
omega ~~ omega #variance
kappa ~~ omega #covariance

#laten vars for AR and cross-lagged effects
p1 =~ 1*x1 #each factor loading set to 1
p2 =~ 1*x2
p3 =~ 1*x3
q1 =~ 1*d1
q2 =~ 1*d2
q3 =~ 1*d3

p3 ~ alpha3*p2 + beta3*q2
p2 ~ alpha2*p1 + beta2*q1

q3 ~ delta3*q2 + gamma3*p2
q2 ~ delta2*q1 + gamma2*p1

p1 ~~ p1 #variance
p2 ~~ u2*p2
p3 ~~ u3*p3
q1 ~~ q1 #variance
q2 ~~ v2*q2
q3 ~~ v3*q3

p1 ~~ q1 #p1 and q1 covariance
p2 ~~ q2 #p2 and q2 covariance
p3 ~~ q3 #p2 and q2 covariance'

fitrecall <- lavaan(riclpmModelrecall, data = Main_varaibles_backup_for_R,
            missing = 'ML', #for the missing data!
            int.ov.free = F,
            int.lv.free = F,
            auto.fix.first = F,
            auto.fix.single = F,
            auto.cov.lv.x = F,
```

```
              auto.cov.y = F,
              auto.var = F)
summary(fitrecall, standardized = T)

standardizedSolution(fitrecall)
#How are the random intercepts distributed?

fscoresrecall <- lavPredict(fitrecall)
library(ggplot2)

fscoresrecall_df <- as.data.frame(fscoresrecall)
fscoresrecall_df$pid <- 1:nrow(fscoresrecall_df)

# Example: plot distribution of RIx
ggplot(fscoresrecall_df, aes(x = kappa)) +
  geom_histogram(bins = 30, fill = "skyblue", color = "black") +
  theme_minimal() +
  labs(title = "Distribution of Random Intercept for X",
       x = "Random Intercept (RIx)", y = "Frequency")

# Example: scatterplot of RIx vs RIy
ggplot(fscoresrecall_df, aes(x = kappa, y = omega)) +
  geom_point(alpha = 0.6) +
  theme_minimal() +
  labs(title = "Random Intercepts: X vs Y", x = "RIx", y = "RIy")

# Now I want to add colours to the scatter depending on how many observation go into the model per participant
library(dplyr)

# Count number of timepoints per pid (any value for x or y at that wave)
obs_count <- Main_varaibles_... %>%
  group_by(pid, wave) %>%
  summarise(has_data = any(!is.na(value)), .groups = "drop") %>%
  group_by(pid) %>%
  summarise(n_timepoints = sum(has_data)) %>%
```

```
  mutate(contribution = case_when(
    n_timepoints == 3 ~ "All 3 time points",
    n_timepoints == 2 ~ "2 time points",
    n_timepoints == 1 ~ "1 time point"
  ))

fscoresrecall <- lavPredict(fitrecall)
fscoresrecall_df <- as.data.frame(fscoresrecall)
fscoresrecall_df$pid <- 1:nrow(fscoresrecall_df)

# Join the contribution info
fscoresrecall_df <- fscoresrecall_df %>%
  left_join(obs_count, by = "pid")



#Scatter
ggplot(fscoresrecall_df, aes(x = kappa, y = omega, color = contribution)) +
  geom_point(alpha = 0.7) +
  theme_minimal() +
  labs(title = "Random Intercepts Colored by Data Contribution",
       x = "RIx", y = "RIy", color = "Data Contribution")
#scatter with correlation line
ggplot(fscoresrecall_df, aes(x = kappa, y = omega, color = contribution)) +
  geom_point(alpha = 0.6) +
  geom_smooth(method = "lm", se = FALSE, color = "black") +
  theme_minimal() +
  labs(title = "Between-Person Association of Random Intercepts",
       x = "RIx (kappa)", y = "RIy (omega)", color = "Data Contribution")



#2.0
ggplot(fscoresrecall_df, aes(x = kappa, y = omega, color = contribution)) +
  geom_point(alpha = 0.7) +
  theme_minimal(base_size = 18) +  # increases overall text size
  labs(
```

```
    title = "Random Intercepts of step count (x) and delayed recall z-score (y)",
    x = "RIx",
    y = "RIy",
    color = "Data Contribution"
  ) +
  theme(
    plot.title = element_text(size = 19, face = "bold", hjust = 0.5),
    axis.title = element_text(size = 20),
    axis.text = element_text(size = 20),
    legend.title = element_text(size = 20),
    legend.text = element_text(size = 20)
  )

#Now the adjusted version
riclpmrecalladjusted <-
  '
#Note, the data contain x1-3 and a1-3
#Latent mean Structure with intercepts

kappa =~ 1*x1 + 1*x2 + 1*x3
omega =~ 1*d1 + 1*d2 + 1*d3

x1 ~ mu1*1 #intercepts
x2 ~ mu2*1
x3 ~ mu3*1
d1 ~ pi1*1
d2 ~ pi2*1
d3 ~ pi3*1

kappa ~~ kappa #variance
omega ~~ omega #variance
kappa ~~ omega #covariance

#laten vars for AR and cross-lagged effects
p1 =~ 1*x1 #each factor loading set to 1
```

```
p2 =~ 1*x2
p3 =~ 1*x3
q1 =~ 1*d1
q2 =~ 1*d2
q3 =~ 1*d3

# Regression of random intercepts on z1
kappa + omega ~ stroke + sex + edu + age +liv

# Add as a sensitivity: Regression on observed variables
 # Regression of observed variables on z1 (constrained)
  #x1 + x2 + x3  ~ s1* sex + age+ stroke + liv + stroke  # Constrained over time.
 # d1 + d2 + d3  ~ s2* sex + age+ stroke + liv +stroke # Constrained over time.

p3 ~ alpha3*p2 + beta3*q2
p2 ~ alpha2*p1 + beta2*q1

q3 ~ delta3*q2 + gamma3*p2
q2 ~ delta2*q1 + gamma2*p1

p1 ~~ p1 #variance
p2 ~~ u2*p2
p3 ~~ u3*p3
q1 ~~ q1 #variance
q2 ~~ v2*q2
q3 ~~ v3*q3

p1 ~~ q1 #p1 and q1 covariance
p2 ~~ q2 #p2 and q2 covariance
p3 ~~ q3 #p2 and q2 covariance'

fitrecalladjusted <- lavaan(riclpmrecalladjusted, data = Main_varaibles_backup_for_R,
                sampling.weights = "ipw_trimmed",
                missing = 'ML', #for the missing data!
                int.ov.free = F,
```

```
                int.lv.free = F,
                auto.fix.first = F,
                auto.fix.single = F,
                auto.cov.lv.x = F,
                auto.cov.y = F,
                auto.var = F)
summary(fitrecalladjusted, standardized = T)

standardizedSolution(fitrecalladjusted)
fitmeasures(fitrecalladjusted)
```

References

1. Wagle J, Selbæk G, Benth J, et al. The CERAD Word List Memory Test: Normative Data Based on a Norwegian Population-Based Sample of Healthy Older Adults 70 Years and Above. The HUNT Study. *J Alzheimers Dis*. 2023;91(1):321–343. doi:10.3233/jad-220672
2. Engedal K, Gjøra L, Benth J, Wagle J, Rønqvist TK, Selbæk G. The Montreal Cognitive Assessment: Normative Data from a Large, Population-Based Sample of Cognitive Healthy Older Adults in Norway-The HUNT Study. *J Alzheimers Dis*. 2022;86(2):589–599. doi:10.3233/jad-215442
3. Borland E, Nägga K, Nilsson PM, Minthon L, Nilsson ED, Palmqvist S. The Montreal Cognitive Assessment: Normative Data from a Large Swedish Population-Based Cohort. *J Alzheimers Dis*. 2017;59(3):893–901. doi:10.3233/jad-170203
4. Luck T, Pabst A, Rodriguez FS, et al. Age-, sex-, and education-specific norms for an extended CERAD Neuropsychological Assessment Battery-Results from the population-based LIFE-Adult-Study. *Neuropsychology*. May 2018;32(4):461–475. doi:10.1037/neu0000440
5. Espenes J, Hessen E, Eliassen IV, et al. Demographically adjusted trail making test norms in a Scandinavian sample from 41 to 84 years. *Clin Neuropsychol*. Dec 2020;34(sup1):110–126. doi:10.1080/13854046.2020.1829068
6. Hooper D, Coughlan J, Mullen M. Structural Equation Modelling: Guidelines for Determining Model Fit. *Electronic Journal of Business Research Methods*. 2008;6(1), 53-60.
7. Austin PC. Balance diagnostics for comparing the distribution of baseline covariates between treatment groups in propensity-score matched samples. *Stat Med*. Nov 10 2009;28(25):3083–107. doi:10.1002/sim.3697
8. Skogseth-Stephani R, Varmdal T, Krizak Halle K, Flø Bjervik T, Haaland krokan TG, Indredavik B. *Norsk hjerneslagregister, Årsrapport for 2023*. 2024. https://www.stolav.no/49f1ce/siteassets/seksjon/norsk-hjerneslagregister/documents/arsrapporter/arsrapport-norsk-hjerneslagregister-2023.pdf

9. Mulder JD, Hamaker EL. Three Extensions of the Random Intercept Cross-Lagged Panel Model. *Structural Equation Modeling: A Multidisciplinary Journal*. 2021/07/04 2021;28(4):638–648. doi:10.1080/10705511.2020.1784738
10. Rosseel Y. lavaan: An R Package for Structural Equation Modeling. *Journal of Statistical Software*. 05/24 2012;48(2):1 – 36. doi:10.18637/jss.v048.i02
